# Decellularised skeletal muscles allow functional muscle regeneration by promoting host cell migration

**DOI:** 10.1038/s41598-018-26371-y

**Published:** 2018-05-30

**Authors:** Anna Urciuolo, Luca Urbani, Silvia Perin, Panagiotis Maghsoudlou, Federico Scottoni, Asllan Gjinovci, Henry Collins-Hooper, Stavros Loukogeorgakis, Athanasios Tyraskis, Silvia Torelli, Elena Germinario, Mario Enrique Alvarez Fallas, Carla Julia-Vilella, Simon Eaton, Bert Blaauw, Ketan Patel, Paolo De Coppi

**Affiliations:** 1 Stem Cells and Regenerative Medicine Section, Great Ormond Street Institute of Child Health, University College London, London, WC1N 1EH UK; 2 Venetian Institute of Molecular Medicine, Via Orus 2, 35128 Padua, Italy; 3 Institute of Hepatology London, Foundation for Liver Research, 111 Coldharbour Lane, London, SE5 9NT UK; 4 Faculty of Life Sciences & Medicine, King’s College London, London, UK; 5 School of Biological Sciences, University of Reading, RG6 6UB Reading, UK; 6 Department of Biomedical Sciences, University of Padova, Padova, Italy; 7 Department of Women’s and Children’s Health, University of Padova, Via Giustiniani 3, 35128 Padova, Italy

## Abstract

Pathological conditions affecting skeletal muscle function may lead to irreversible volumetric muscle loss (VML). Therapeutic approaches involving acellular matrices represent an emerging and promising strategy to promote regeneration of skeletal muscle following injury. Here we investigated the ability of three different decellularised skeletal muscle scaffolds to support muscle regeneration in a xenogeneic immune-competent model of VML, in which the EDL muscle was surgically resected. All implanted acellular matrices, used to replace the resected muscles, were able to generate functional artificial muscles by promoting host myogenic cell migration and differentiation, as well as nervous fibres, vascular networks, and satellite cell (SC) homing. However, acellular tissue mainly composed of extracellular matrix (ECM) allowed better myofibre three-dimensional (3D) organization and the restoration of SC pool, when compared to scaffolds which also preserved muscular cytoskeletal structures. Finally, we showed that fibroblasts are indispensable to promote efficient migration and myogenesis by muscle stem cells across the scaffolds *in vitro*. This data strongly support the use of xenogeneic acellular muscles as device to treat VML conditions in absence of donor cell implementation, as well as *in vitro* model for studying cell interplay during myogenesis.

## Introduction

Skeletal muscle is the most abundant tissue in the human body and composed of muscle fibres, muscle stem cells, nerves, blood vessels, interstitial cells and ECM. Skeletal muscle regeneration is dependent on SCs, the resident stem cells of muscle located beneath the basal lamina of muscle fibres^1–3^. Despite having regenerative ability, skeletal muscle is unable to recover when the defect is too extensive (e.g. congenital malformations, traumatic injuries, surgical ablations or degenerative myopathies). As a consequence, skeletal muscle is not able to replace a VML and the result is a modification of the tissue architecture and composition accompanied by fibrosis and subsequent functional impairment or loss^4^. Available approaches to treat VML damages do not allow functional recovery of the damaged muscle^5^. Therefore, there is a great demand for developing new therapeutic strategy for VML.

Recent studies have shown the crucial role played by 3D environment and ECM on regulating stem cells identity and function^6^. Bioengineering approaches have attempted to combine natural/synthetic scaffolds with stem cells and growth factors for application in regenerative medicine^7^. Biomaterials have to replicate the properties of tissue-specific ECM, providing a 3D scaffold where stem cells can preserve their identity, adhere, proliferate, differentiate and generate a cellular 3D structure resembling the tissue of interest. Moreover, it is also important that scaffolds have a good rate of biocompatibility and biodegradability in order to promote progressive replacement with newly formed tissue without inducing any adverse inflammatory response, which could lead to scar tissue formation or scaffold rejection after *in vivo* implantation^5^. Despite improvement in biomaterials’ fabrication in recent years, there is an unmet need to develop scaffolds that respect all the above characteristics and support the development of functional tissues^8,9^. Generation of ECM scaffolds by means of decellularisation eliminates cellular and nuclear content, but maintains biological activity, mechanical integrity and 3D structure of the tissue from which the ECM is derived^5^. Commonly used methods of decellularisation include the use of chemical or enzymatic agents and physical methods such as sonication^10^. Acellular scaffolds are biocompatible and are not rejected after allogeneic or xenogeneic transplantation^5^. A number of studies have successfully obtained acellular scaffolds from organs such as trachea^11^, heart^12^, kidney^13^, pancreas^14,15^, lung^16,17^, liver^18,19^ and intestine^20^. Indeed, some decellularised organs are in clinical use^21–23^.

Acellular tissues –such as pig urinary bladder ECM, have been clinically used to treat VML conditions^24^, and only recently acellular skeletal muscle matrices have been tested for the same application in animal model of VML^25–27^. However, it still remains a matter of discussion whether the final *in vivo* outcome of acellular tissues can be influenced by the original tissue from which they are derived and by the specific protocol used for the decellularisation^5,28–30^. Here we investigate the ability of xenogeneic acellular muscles derived with three different perfusion protocols of decellularisation to be used as a device to promote functional muscle regeneration without the implementation of donor cells. We showed that once implanted in a murine model of VML to replace a resected muscle, acellular scaffolds permit the development of an artificial muscle able to contract and generate force. Preservation of ECM components and 3D topology was the sufficient requirement to drive host cells toward scaffold repopulation, which allowed proper muscular stem cell maintenance, cell differentiation and homing, as well as functional tissue formation.

## Methods

### Animals

All the procedures performed on animals were in accordance with the Home Office and all the experimental protocols were approved by the UK Home Office, Project Licence PPL 70/7622. 250–350 g male or female Sprague Dawley rats were used for acellular muscle preparation. 3–4 months old C57BL/6J mice were used for *in vivo* scaffold implantation. C57BL/6J mice and transgenic GFP+ or transgenic C57BL/6-(ACTB-EGFP)/J mice were used a source of muscle stem cells (SC) and fibroblasts (FB). Mice were housed in individual cages in an environmentally controlled room (23 °C, 12 h light/12 h dark cycle) and provided food and water ad libitum.

### Dissection of rat lower limb

250–350 g rats were used as a source of muscle for decellularisation. Rats were killed by CO_2_ inhalation and death confirmed by onset of *rigor mortis*, the skin was carefully removed from the lower limb, abdomen, and lower back on the side of the desired limb (distally to the ankle does not need to be skinned, and may harm vasculature in doing so). In order to gain access to the iliac vessels, the abdomen was opened and bowels were resected. A 24 G cannula was inserted into the abdominal iliac artery and advanced distally. A suture was placed tightly around the femoral vessels to secure the cannula distal to the reflection of the abdominal wall and the major branches to the genitalia. On the medial aspect, half way between the hip and the knee, large cutaneous branches were also ligated. The leg was dissected from the rest of the body by splitting the pelvis at the pubic symphysis and the sacroiliac joint. Muscles of the lower back and the iliopsoas were dissected, to complete separation, sparing damage to the muscles of the lower limb.

### Decellularisation protocols

The three protocols used, based on published studies^29–33^, were adapted to a perfusion condition and are summarized in Table 1. Perfusion of all solutions was achieved with a peristaltic pump (Rating Plate- iPump i150, iPump) at a flow rate of 1 ml/min. Optimal cycle number and conditions were determined by assessing DNA removal and preservation of tissue architecture.

**Table 1:** Summary of the three decellularisation protocols.

LatB (1 cycle)			DET (3 cycles)			SDS (1 cycle)		
Time (h)	T (°C)	Reagent	Time (h)	T (°C)	Reagent	Time (h)	T (°C)	Reagent
2	37	50 nM Latrunculin B in high glucose DMEM	ON	4	de-ionized water	72	RT	0.25% SDS
½	RT	wash with PBS	4	RT	4% sodium deoxycholate	48	RT	wash with de-ionized water
2	RT	0.6 M potassium chloride	½	RT	wash with PBS			
2	RT	1.0 M potassium iodide	3	RT	34 kU/ml DNase in 1 M NaCl			
ON	RT	de-ionized water	48	RT	de-ionized water			
2	RT	0.6 M potassium chloride						
2	RT	1.0 M potassium iodide						
2	RT	34 kU/ml DNase in 1 M NaCl						
ON	RT	de-ionized water wash						

LatB, Latrunculin B protocol; DET, Detergent Enzymatic Treatment; SDS, Sodium Dodecyl Sulfate protocol; ON, over night; T, temperature; RT, room temperature.

Latrunculin B protocol (LatB): limbs were perfused with 50 nM latrunculin B (Sigma-Aldrich) in high-glucose Dulbecco’s modified Eagle’s medium (DMEM) (Gibco, Life Technologies, USA) for 2 hours at 37 °C. All of the following steps were performed at room temperature; washed with PBS for 30 minutes, 0.6 M potassium chloride (Sigma) for 2 hours, 1.0 M potassium iodide (Sigma) for 2 hours, de-ionized water overnight. The following day salt solutions were repeated, followed by perfusion with DNase-I (Sigma) in 1 M NaCl (Sigma) for 2 hours and overnight de-ionized water wash.

Each cycle of Detergent Enzymatic Treatment (DET) consisted of perfusion with de-ionised water (resistivity 18.2 MΩ/cm) at 4 °C overnight, 4% sodium deoxycholate (Sigma) at room temperature (RT) for 4 hours, wash with PBS for 30 minutes, and 34 kU/ml DNase-I (Sigma) in 1 M NaCl (Sigma) at RT for 3 hours, and a final wash with de-ionized water for 48 hours.

Sodium Dodecyl Sulfate protocol (SDS): limbs were perfused with 0.25% SDS (Sigma) for 72 hours and washed in de-ionized water for 48 hours.

After each decellularisation treatment, muscles of interest were dissected and preserved at 4 °C, in PBS with 1% Penicillin/Streptomycin (P/S - Gibco).

### DNA quantification and agarose gel electrophoresis

DNA was extracted from tissues using DNA extraction kit (PureLink Genomic DNA MiniKit, Invitrogen) according to the manufacturer’s instructions. DNA content was estimated using a spectrophotometer (Tecan Infinity), measuring optical densities at 260 nm and 280 nm.

### Histology and Immunofluorescence

Samples were fixed in 10% neutral buffered formalin (pH 7.4), dehydrated through an alcohol series, embedded in paraffin and sectioned at a thickness of 5 μm. Tissue slides were stained with haematoxylin and eosin (H&E), Masson’s trichrome (MT), picrosirius red (PR) or elastin Van Gieson (EVG; all Leica, Raymond A Lamb, BDH Chemicals Ltd) stains. For cryosections (10 μm), samples were fixed in PFA 4% in PBS for 10 mins at room temperature before being processed for the staining. Samples were analysed with an Olympus BX60 light microscope equipped with Olympus DP50 digital camera. Myofibre cross sectional area was calculated on 10x images by using ImageJ software; at least 500 myofibres were evaluated for each sample. For immunofluorescence analysis of acellular muscles, slides were de-paraffinized, antigen retrieval was performed using 10 mM sodium citrate buffer (pH 6.0) at 95 °C for 20 minutes, treated with 0.25% Triton X100 for 90 minutes at room temperature, and non-specific antibody binding was blocked with 5% horse serum in PBS for 1 hour at room temperature. Primary antibodies were added and left overnight at 4 °C; antibodies used were rabbit anti rat laminin (Abcam), at a dilution of 1:150; anti rat collagen IV (Abcam), at a dilution of 1:100; anti collagen I (Abcam), at a dilution of 1:100; anti dystrophin (Abcam), at a dilution of 1:200; anti α-dystroglycan (Millipore), at a dilution of 1:200; anti β-dystroglycan (Leica Biosystems), at a dilution of 1:50. Washes were performed in PBS and an Alexa Fluor® 594 Goat Anti-Rabbit IgG (Invitrogen) was used as a secondary antibody at a dilution of 1:150. Washes were repeated and a mounting medium with DAPI (Vector Laboratories, USA) was used to secure the cover-slip. For immunofluorescence analysis of mouse EDL muscles, implants and *in vitro* repopulated scaffolds, samples were snap frozen and cross-sections or longitudinal sections of 10 μm were prepared. To characterize primary cells, cells were firstly fixed in PFA at 4% in PBS, 5 minutes at room temperature. Cryo-sections or cells were then incubated for 1 hr at room temperature with 10% fetal bovine serum dissolved in PBS 0.1% Triton X100. When mouse antibodies were used for *ex vivo* samples, MOM kit (Vector laboratories) was used for the blocking step. Samples were then incubated with primary antibodies at 4 °C overnight. The following primary antibodies were used: mouse anti-Tubulin β3 (Clone Tuj1) (1:500, Biolegend); rabbit anti-laminin (1:150, Sigma); rabbit anti-von Willebrand factor (1:100, Abcam); rabbit anti-synaptophysin (1:100, Sigma); mouse anti-myosin heavy chain (1:100, R&D); mouse anti-embryonic myosin heavy chain (1:25, Hybridoma Bank); rabbit anti-ki67 (1:80, Abcam); mouse anti-pax7 (1:25, Hybridoma Bank); rabbit anti-α-smooth muscle actin (α-SMA, 1:100, Abcam); anti-desmin (1:100; Abcam); anti-vimentin (1:100; Abcam); rabbit anti-MyoD (1:50, Santa Cruz); anti-tcf4 (1:100; Millipore); mouse anti-MyoG (1:50, Abcam). After washings, samples were incubated with the appropriate secondary antibody for 1 hr at room temperature. Secondary antibodies used were anti-mouse (1:200, Invitrogen), anti-rabbit (1:200, Invitrogen), anti-rat (1:200, Sigma). For triple myosin and laminin co-staining, primary antibodies used were: BA-D5 (IgG 2b, 1:100, DSHB supernatant), SC71 (IgG1, 1:100 DSHB, supernatant), BF-F3 (IgM, 1:100 DSHB, purified antibody), anti-laminin (1:100, Sigma-Aldrich), prepared in PBS-BSA 1%. Secondary antibodies were: DyLight 405 labeled goat anti mouse IgG, Fcγ 2b subclass specific (Jackson ImmunoResearch Europe), 1:200, Alexa Fluor 488 labeled goat anti mouse IgG, Fcγ 1 subclass specific (Jackson ImmunoResearch Europe) 1:200, Alexa Fluor 594 of Cy3 labeled goat anti mouse IgM, µ chain specific (Jackson ImmunoResearch Europe) 1:200, Alexa Fluor 647 labeled goat anti rabbit IgG (111-604-144, Jackson ImmunoResearch Europe) diluted in PBS-BSA 1%. Nuclei were stained with Hoechst 33258 (Sigma) dissolved in secondary antibody mix. When bungarotoxin staining was performed, tetramethylrhodamine conjugate α-bungarotoxin (Invitrogen) was added in the secondary antibody mix. Samples were analysed with a Leica DMI6000B fluorescence microscope with motorized stage or with a Leica SP5 confocal microscope.

### Collagen and GAG quantification

Collagen was quantified using a QuickZyme Total Collagen Kit (2B Scientific, UK). Quantity of sulphated glycosaminoglycans (GAG) was measured using a Blyscan GAG Assay Kit (Biocolor, UK).

### Scanning Electron Microscopy (SEM)

Decellularised and fresh samples were fixed in 2.5% glutaraldehyde in 0.1 M phosphate buffer for 24 hours at 4 °C, followed by washes in 0.1 M phosphate buffer. The samples were then cut onto segments of approximately 1 cm in length and immersed in a cryoprotective solution of 25% sucrose, 10% glycerol in 0.05 M phosphate buffer (pH 7.4) for 2 hours, then fast frozen and fractured in a nitrogen slush of approximately −160 °C, replaced in the cryoprotective solution at room temperature and allowed to thaw. The samples were then washed in 0.1 M phosphate buffer (pH 7.4), fixed in 1% OsO_4_/0.1 M phosphate buffer (pH 7.3) at 3 °C for 1.5 hours, and washed again in 0.1 M phosphate buffer. Samples were then rinsed in deionized water and dehydrated using ethanol, critical point dried using CO_2_ and mounted on aluminium stubs, presenting the fractured surface of the samples. This surface was then coated with a thin layer of Au/Pd (approximately 2 nm) using a Gatan ion beam coater. Images were taken using a Jeol 7401 FEG scanning electron microscope.

For single fresh myofibres, anucleate and seeded ones, were fixed in 4% PFA for 10 minutes, dehydrated through 30, 50, 70, 80, 90, and 100% ethanol solutions (15 minutes for each step) and transferred to a critical point drier (Balzers CPD 030—using liquid carbon dioxide). Dried myofibres were transferred to SEM chucks using microforceps under a light microscope. Thereafter, myofibres were gold-coated using an Edwards S150B sputter-coater and examined using a FEI 600 F scanning electron microscope aided by analysis software for image collection.

### Protein extraction and ELISA

ELISAs were performed for VEGF and IGF-I using Quantikine Immunoassay kits (RV00 and MG100 respectively) (R&D systems, USA).

The ELISA was performed according the manufacturer instructions. Optical density was measured using a Fluostar Optima microplate reader (BMG Labtech, Germany) at 450 nm wavelength with a correction wavelength of 570 nm.

### Chick chorioallantoic membrane (CAM) angiogenic assay

Fertilized chicken eggs (Henry Stewart and Co., UK) were used to assess the angiogenic capabilities of the decellularised scaffolds. Eggs were incubated at 37 °C in controlled humidity; on day 3 oval windows of approximately 3 cm in diameter were made in the shell, thus exposing the embryo and CAM vessels. The window was then resealed with tape and replaced in the incubator for a further 5 days. At this point, samples of decellularised scaffolds approximately 1.5 mm side length and square shaped were placed on the CAM between visible branches of vessels. Polyester sections soaked overnight either in a PBS solution or in PBS with 200 ng/mL VEGF were used as negative and positive controls respectively. On day 0, 5 and 7 after implantation the samples were photographed in ovo. The number of blood vessels converging towards the placed tissues was counted blindly by assessors (n = 4), with the mean of the counts being considered. Moreover, cell infiltration and vessels formation into the scaffolds were also assess by staining cryosections with Alexa Fluor® 594 Phalloidin (Molecular Probes, USA) and nuclei with mounting medium with DAPI (Vector Laboratories, USA).

### Extensor digitorum longus (EDL) ablation and scaffold implantation

Mice were anaesthetized using 0.5–3% isoflurane with oxygen via a nose cone. Induction of deep surgical anaesthesia was confirmed by absence of reaction to hind foot pinch. The surgical procedure underwent in aseptic conditions (skin preparation, sterilized instruments, gloves and drapes). It started with a skin incision along the tibia length then going lateral to the proximal edge of the Tibialis Anterior (TA) of the right leg. Subsequently, after the opening of the aponeurosis, TA was gently moved to expose the EDL and the distal tendon of EDL was identified and transected. The EDL was then everted along its whole length and the first anastomosis, between one edge of the scaffold and the native EDL at half a centimetre from the proximal tendon, was made with a non-absorbable Prolene 7–0 suture. A second anastomosis, joining the other edge of the scaffold and distal tendon of native EDL, was made with a Prolene 7-0 suture. The native EDL was then resected between the anastomoses. Finally, the implanted EDL was repositioned in its original anatomic position and the skin wound was closed with Vicryl 6-0 suture. In the control EDL ablation’s mice (sham), the surgical procedure was the same, but no scaffolds were implanted; the native EDL was cut at the distal tendon and half a centimetre from the proximal tendon. Implanted scaffolds were derived from rat EDL acellular muscles, and were finely cut along the major axis to obtain scaffolds of the right size. No movement or space restriction were applied to animals after treatments.

### *Ex vivo* force measurement

Two months after implantation, animals were killed by CO_2_ inhalation and cervical dislocation. Implants and EDL muscles of contralateral limbs were dissected and used to measure force production *ex vivo*. Force production of EDL muscles was determined as described previously^34^.

### Primary cell preparation and culture into acellular muscles

Mice were sacrificed by CO_2_ inhalation and cervical dislocation, according to UK legal guidelines. Single myofibres isolated from EDL muscles of three-month old wild-type C57BL/6J mice (wt) were the source of SCs and FBs for seeding into decellularised muscles. Single fibres were isolated from EDL muscles as previously described^35^. To release SCs from the myofibres, single fibre preparation was then triturated 21 times using an 18 G needle (MicrolanceTM, US) mounted onto a 1 ml syringe, filtered through a 40 µm cell strainer and plated into a 60 × 20 mm culture-dish previously coated with 0.1% gelatine for 2 hours. SCs were cultured and expanded in proliferating medium (DMEM high glucose, 20% HS, 10% FBS, 1% P/S, 1% L-Glu, 1% Chicken Embryo Extract (CEE; Seralab, UK) and 0.5 µl/ml basic Fibroblast Growth Factor (bFGF; Sigma) at maximum for 1 passage, and then seeded into the scaffolds (expanded SCs were named MuSCs). FBs were isolated from mouse EDL harvested for single fibres preparation and not used for SC isolation (i.e. discarded tissue derived from the single fibre prep). FBs were expanded in DMEM, 20% FBS, 1% P/S, 1% L-Glu for a minimum of 3 passages before seeding into the scaffolds. Wild type- and GFP-MuSCs for seeding experiments on enucleated fibres were isolated from three-month old wild-type C57BL/6J mice (wt) and transgenic C57BL/6-(ACTB-EGFP)/J mice (GFP), with the expression of the enhanced GFP under the control of chicken beta actin promoter, as described in Pasut *et al*.^36^. Hindlimb muscles were dissected and carefully minced. Enzymatic digestion was performed with collagenase-dispase solution (2.5 U/mL) at 37 °C for 12 min. This step was repeated until the tissue was almost entirely digested and appeared as a homogenous solution with no tissue chunks. An equal volume of 10% FBS-DMEM supplemented with pen strep was added and the cell suspension was filtered through a 70 μm filter. After centrifugation at 1800 rpm for 5 min, the supernatant was discarded and cells resuspended in fresh SC growth medium (Ham’s F10 media, 20% FBS, 1% Pens/Strep, 2.5 ng/mL bFGF). Cells were pre-plated in petri dishes coated with 0.01% collagen type I (Corning) for 1 hour and the supernatant transferred in freshly coated petri dishes. This step was repeated other 2 times with pre-plating intervals of 24 hours before using the MuSCs for expansion and seeding. Acellular scaffold for cell seeding measured 8–10 mm length were obtained from decellularised rat hindlimb muscles. MuSCs, FBs or a mix of MuSCs and FBs (70:30) were suspended in a total amount of 5 µl of proliferating medium and injected into the scaffolds under a stereomicroscope and by using an insulin syringe. Cells were either seeded at a density of 1 × 10^6^ cells per scaffold using multiple injections (for long term cultures) or 3 × 10^5^ cells per scaffold with a single injection (for migration assay). Then seeded scaffolds were cultured in cell incubator for 7 or 14 days. During the first two days of culture, samples were cultured in DMED, 20% HS, 10% FBS, 1%P/S, 1% L-Glu, 1% CEE, 0.5 µl/ml bFGF. After 48 hours the medium was changed with DMEM, 2% HS, 1% P/S, 1% L-Glu until the end of the experiment. Single anucleate fibres were isolated from the EDL muscle of decellularised rat hindlimbs. Undamaged EDL muscle was dissected with both tendons intact and incubated with 0.1% collagenase type I in DMEM at 37 °C 5% CO_2_ for 2 hours. Using glass pipettes single anucleate fibres were collected and plated out in culture dishes containing PBS. Anucleate myofibres were gently washed in PBS 3 times before seeding or fixation. MuSCs were seeded on single anucleate myofibres in 12-multiwell plates without coating and monitored using phase-contrast microscope systems that harbour controlled-environment chambers maintained at 37 °C, supplemented with 5% CO_2_ as previously described^37^. Single freshly isolated myofibres from wt mouse-EDL muscles were used as control. Myofibres were cultured in wells containing DMEM supplemented with 10% FBS and 0.5% CEE. Time-lapse video was taken at rate of one frame every 15 minutes for periods of up to 24 hours using an x10 objective. Time-lapse experiments were performed with GFP-MuSCs. In a separate analysis, high power time-lapse microscopy using an x100 objective was carried out, enabling the visualization of cell surface blebbing on single MuSCs. For such analysis, cells were cultured for 24–48 hours, then selected, and subsequently filmed at a rate of one frame per 2 seconds. All image analysis was carried out using ImageJ (version 1.4.3). MuSCs were individually manually tracked using the plugin MTrackJ, and bleb dynamics were quantified using ImageJ. Bleb number was quantified manually on SEM images of cells using Adobe Photoshop version CS2, only SEM images of whole cells were used for quantification. MuSCs differentiation profiles following differing culture conditions were manually assessed using quantification of live images through the Zeiss Axioscope and Axiovision digital camera system. Only cells adherent to the myofibres were considered for the quantification. Statistical analysis was performed using Student’s t test unless indicated otherwise with a significance level of p < 0.05.

### MTT analysis

MTT cell proliferation assay was performed on seeded scaffolds to determine the vitality and proliferation of seeded cells after 7 days of culture using Vybrant® MTT cell proliferation assay kit (Life Technologies; Thermo Fisher scientific). Images of the whole scaffolds were taken with a stereomicroscope (Leica) and then samples were dissolved each in 500 μl of propan-2-olo acid solution overnight in agitation and at room temperature. For MTT quantification, scaffolds were removed and the absorbance of the isopropanol was recorded at 555 nm with a plate reader (Infinite F200, Tecan).

### Statistical analysis

For immunofluorescence and immunohistochemical analyses, at least 8 random high-power field areas were considered per each condition analysed. We expressed data as means ± s.e.m. or, where indicated, as means ± s.d. We determined statistical significance by one-way ANOVA and Tukey’s multiple comparison test or Kruskal-Wallis and Dunn’s multiple comparison test. A p value of less than 0.05 was considered statistically significant. Where not differently specified, n refers to the number of animal/scaffolds analysed.

### Data availability

The datasets generated during and/or analysed during the current study are available from the corresponding author on reasonable request.

## Results

### Intravascular decellularisation of rat hindlimb allowed generation of acellular skeletal muscle tissue

**Figure 1:**
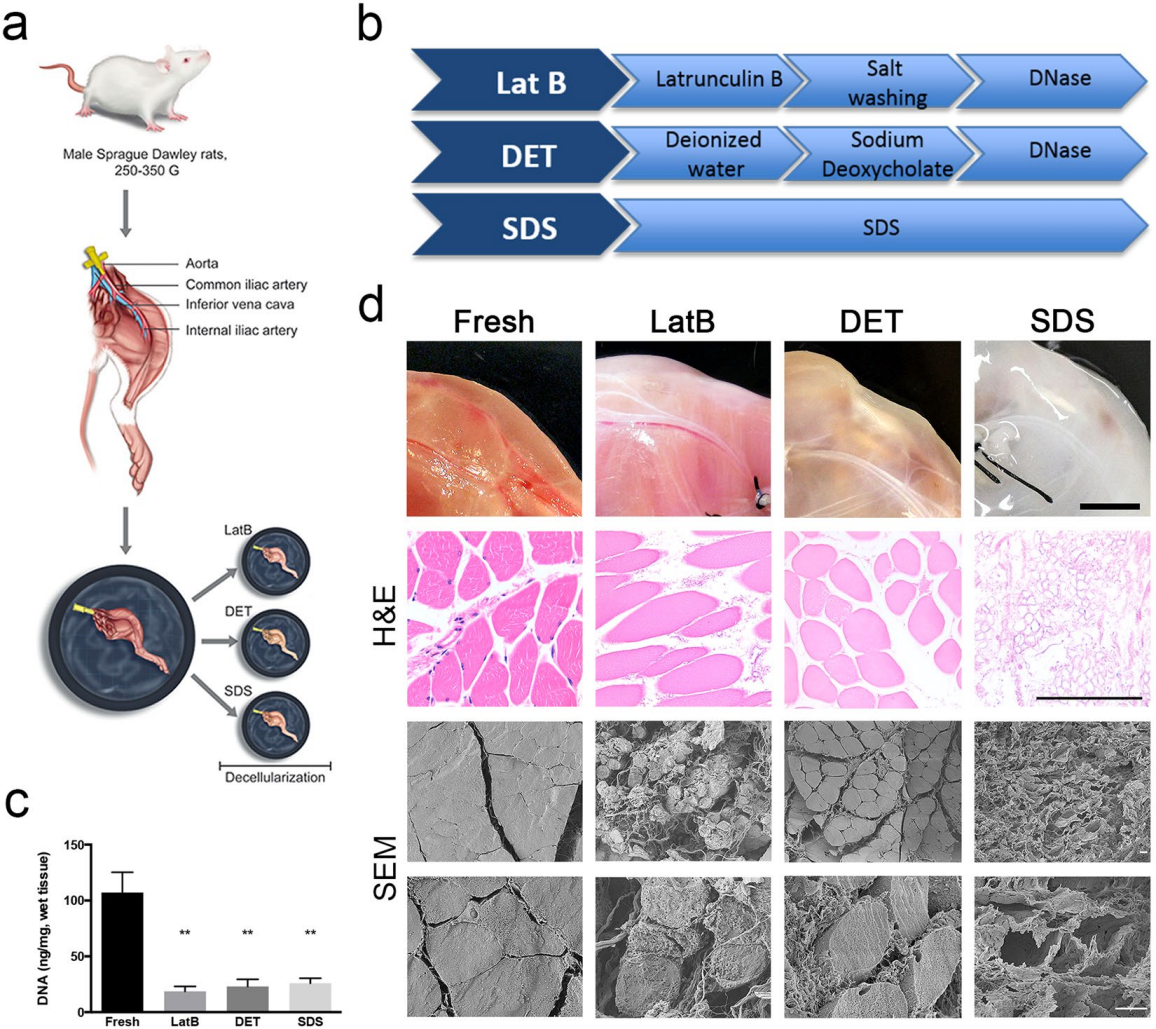
Decellularisation protocols. (**a**) Diagram showing the process used to prepare rat hind limbs for decellularisation. The femoral artery is cannulated and the hind limb dissected; decellularised reagents where delivered through the vessel tree by using a pump directly connected to the cannula. Three different decellularisation methods were performed, and called LatB, DET and SDS. (**b**) Diagram showing steps used to perform LatB, DET and SDS decellularisation. (**c**) Quantification of DNA content in freshly isolated rat skeletal muscle and LatB-, DET- and SDS-decellularised muscles. Data are shown as mean ± s.e.m of three independent replicates; **P < 0.01, one-way ANOVA and Tukey’s multiple comparison test; no significant differences were observed among the decellularised samples; n = 6–10, each group. (**d**) Macroscopic and microscopic (H&E and scanning electron microscopy, SEM) evaluation of fresh rat muscles and LatB-, DET- and SDS-decellularised samples. For H&E analysis, cross-sections were analysed. Scale bars, macroscopic: 1 cm; H&E: 100 µm; SEM: 10 µm.

To allow homogenous and good level of decellularisation of thick skeletal muscle tissue from rat hind limb, constant perfusion of decellularising solutions through the muscle vessels was performed^26^. Acellular scaffolds were created using three different decellularisation protocols: LatB, DET and SDS (Fig. 1a,b). For LatB, one cycle was sufficient to remove the cellular compartment and preserve the ECM structure, whilst three cycles for DET and 72 hours of SDS perfusion were needed to obtain the same level of decellularisation (Supplementary Fig. 1). Treated hind limbs displayed different macroscopic appearance (Fig. 1d). Compared to freshly isolated muscles, DNA content was significantly reduced by all decellularisation protocols (Fig. 1c), a finding confirmed by H&E staining and scanning electron microscopy (SEM). However, both H&E and SEM analyses clearly showed that, among the 3 protocols, acellular muscles displayed different histological characteristics. General tissue structure including myofibres was largely preserved in LatB- and DET-generated samples. In contrast, myofibre content was greatly depleted by SDS treatment leaving mainly the ECM (Fig. 1d).

### Native skeletal muscle components were differently preserved among the three decellularisation protocols

**Figure 2:**
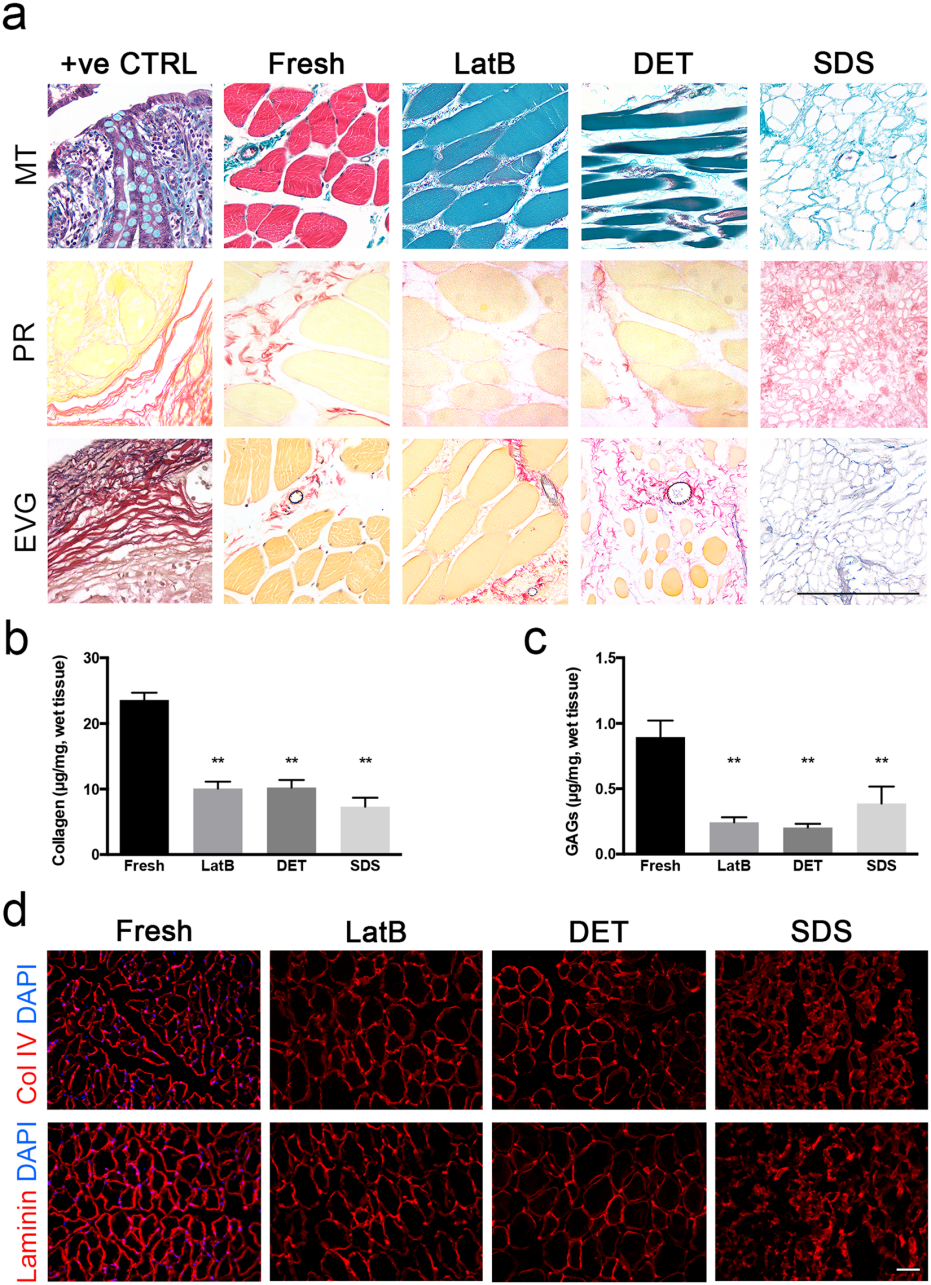
Characterization of decellularised skeletal muscle ECM. (**a**) Masson’s trichrome (MT), picrosirius red (PR) and elastin Van Gieson (EVG) staining of cross-sections from positive control tissues (+ve CTRL), freshly isolated rat skeletal muscle (fresh), LatB-, DET- and SDS-acellular muscles. Scale bar, 10 µm. (**b**) Collagen quantification of freshly isolated rat skeletal muscle (fresh), LatB-, DET- and SDS-acellular muscles. (**c**) GAGs quantification of freshly isolated rat skeletal muscle (fresh), LatB-, DET- and SDS-acellular muscles. Data are shown as mean ± s.e.m of three independent replicates; **P < 0.01; one-way ANOVA and Tukey’s multiple comparison test; no significant differences were observed among the decellularised samples; n = 6–10, each group. (**d**) Representative images of immunofluorescence analysis for collagen IV (Col IV) and laminin (all red) in cross-sections derived from freshly isolated rat skeletal muscle (fresh), LatB-, DET- and SDS-acellular muscles. Nuclei were stained with dapi (blue). Scale bar: 100 µm.

To better understand how the three decellularisation protocols affected key proteins of skeletal muscle homeostasis, a series of specific histological staining were performed on cross sections of freshly isolated rat skeletal muscle and LatB-, DET- and SDS-acellular muscles. Masson’s trichrome (MT), picrosirius red (PR) and elastin Van Gieson (EVG) were used to evaluate the nuclei, cytoplasm and ECM maintenance upon decellularisation (Fig. 2a). All the staining confirmed absence of nuclei in decellularised tissues. MT and PR showed that collagen was still present in all acellular muscles. EVG staining showed the maintenance of elastin in all the derived-acellular muscles (Fig. 2a). All decellularised muscles contained significantly less collagen and GAG than freshly isolated muscles (Fig. 2b,c). Examination of specific ECM components revealed the presence of collagen I, collagen IV and laminin (Fig. 2d, and Supplementary Fig. 2). In contrast, specific sarcolemma proteins were differentially preserved depending on the method used for decellularisation. In particular, dystrophin was almost completely lost in all the scaffolds with some fibers still positively stained in LatB- acellular muscles (Supplementary Fig. 2). Instead α- and β-dystroglycans were almost completely lost in DET-decellularised muscles, but partially preserved in those generated by LatB- and SDS (Supplementary Fig. 2).

### Acellular scaffolds promoted angiogenesis *in vivo* and preserved chemokines

**Figure 3:**
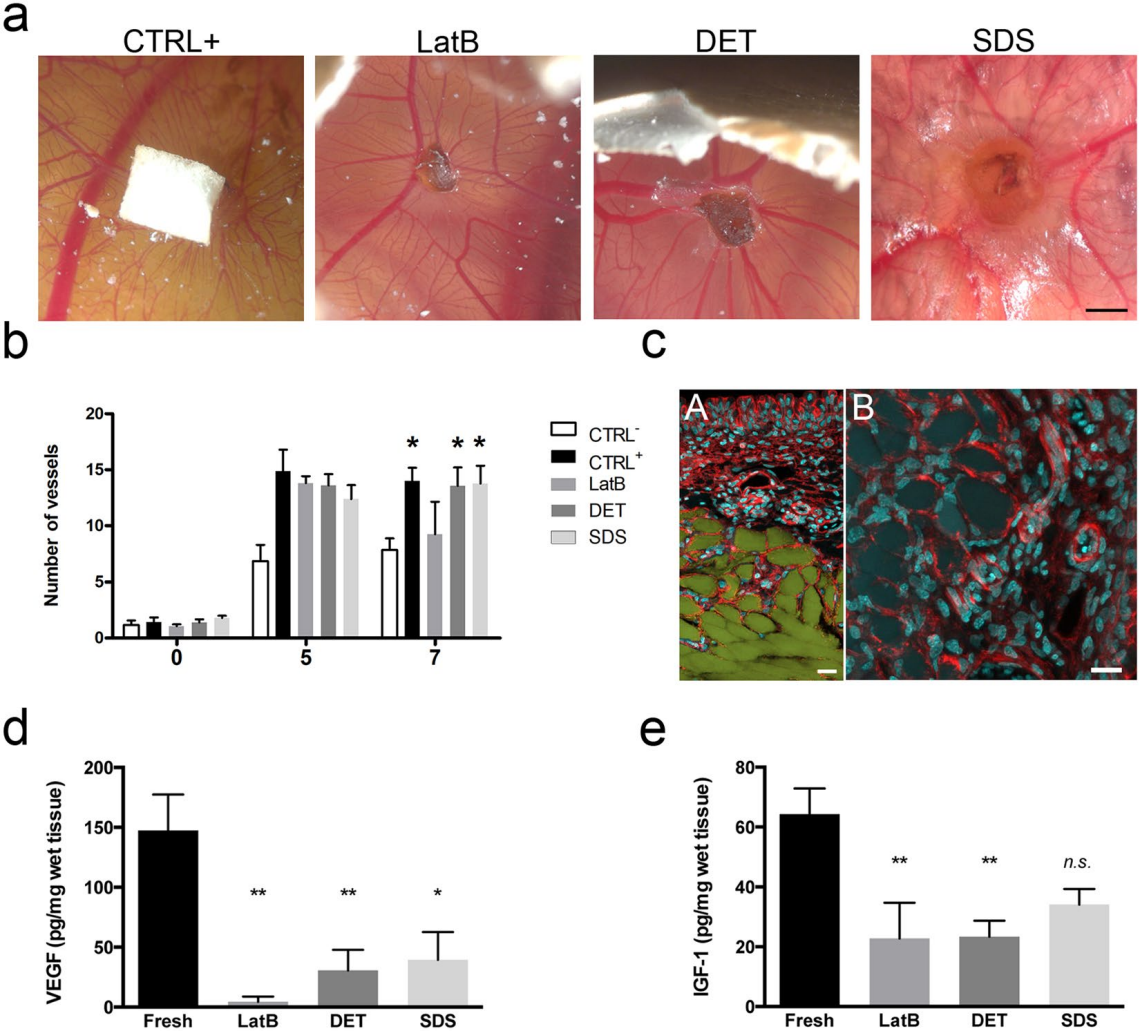
Angiogenetic properties and chemokine maintenance of decellularised skeletal muscles. (**a**) Representative images of CAM assay performed for filter embedded with VEGF (positive control, CTRL+), and LatB-, DET- and SDS-acellular muscles. Scale bar: 2 cm. (**b**) Quantification of vessel converged toward filter loaded with PBS (negative control, CTRL-) or VEGF (positive control, CTRL+) and LatB-, DET- and SDS-acellular muscles at the moment of the implantation (0), 5 or 7 days after implantation. The number of vessels significantly increased 7 days after implantation (0) in CTRL+, DET and SDS samples. Data are shown as mean ± s.e.m of three independent replicates; no significant differences were observed among the three scaffolds and positive control; n = 4 independent experiment per each group. (**c**) Representative images of DET-acellular muscle cryosections 7 days after egg implantation, and stained with phalloidin (red) and dapi (blue). Autofluorescence of the scaffold (green) is also showed in panel A. Panel B, higher magnification of phalloidin (red) and dapi (blue). Scale bar: 25 µm. (**d**) Quantification of VEGF content in freshly isolated rat skeletal muscle and LatB-, DET- and SDS-decellularised muscles. Data are shown as mean ± s.e.m of three independent replicates; *P < 0.05; **P < 0.01, each decellularised sample compared to fresh muscles; one-way ANOVA and Tukey’s multiple comparison test; no significant differences were observed among the decellularised samples; n = 6–12, each group. (**e**) Quantification of IGF-1 content in freshly isolated skeletal rat muscles and LatB-, DET- and SDS-decellularised muscles. Data are shown as mean ± s.e.m of three independent replicates; **P < 0.01, each decellularised sample compared to fresh muscles; n.s., not significant; one-way ANOVA and Tukey’s multiple comparison test; no significant differences were observed among the decellularised samples; n = 4–8, each group.

We next determined whether acellular muscles would support *in vivo* angiogenesis using an established CAM assay (Fig. 3a–c). The number of vessels converging towards the decellularised samples DET and SDS was identical to the positive control and higher than the negative control (Fig. 3a,b), with evidence of cell and vessel invasion into the scaffolds (Fig. 3c). ELISA analysis confirmed the presence of relevant chemokines in the decellularised muscles. In particular, vascular endothelial growth factor (VEGF) was still detectable in DET- and SDS-acellular muscles, albeit reduced when compared to freshly isolate muscles (Fig. 3d). In contrast, LatB-scaffolds showed complete loss of VEGF (Fig. 3d) and a lower angiogenic effect in the CAM assay. Moreover, all the three decellularised procedures preserved the insulin-like growth factor (IGF-1), despite its amount was reduced when compared to freshly isolated muscles (Fig. 3e).

### Acellular scaffolds restore muscle function in a murine model of VML

**Figure 4:**
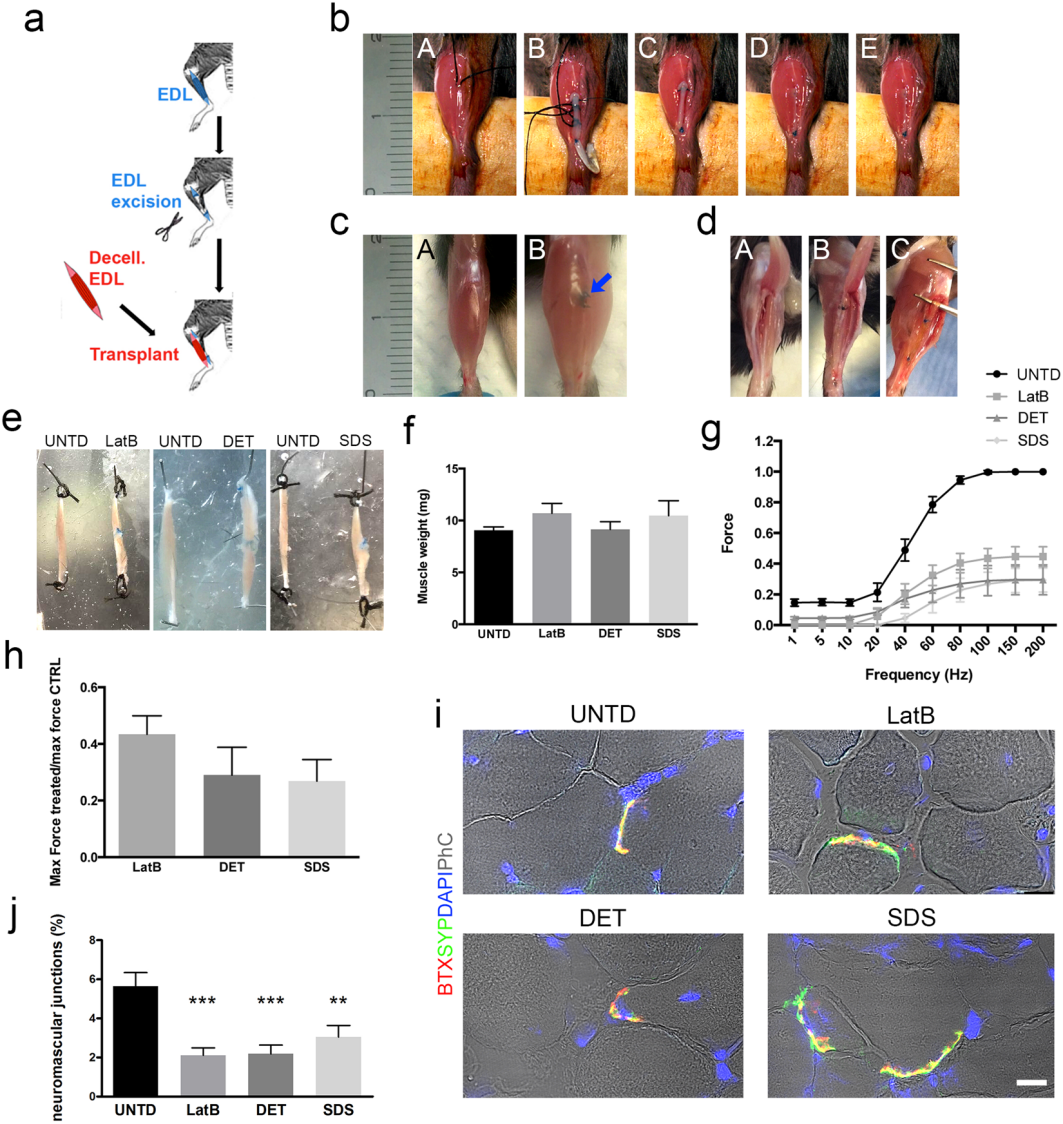
Implanted decellularised skeletal muscles allow functional muscle regeneration in a mouse model of VML. (**a**) Schematic diagram showing the surgical strategy used to generate VML model and implantation of acellular scaffolds. (**b**) Representative images showing the surgical procedure used for scaffold implantation. EDL muscle was exposed (A) and a first anastomosis was performed between the proximal edge of EDL muscle and the scaffold (B). The second anastomosis was carried out to the distal tendon of the EDL, then the scaffold length adapted to the implant size and the native EDL muscle resected between the two anastomosis (C). No-absorbable stitches are blue. Implant was relocated in the original anatomic position (D) and skin sutured (E). (**c**) Representative images showing macroscopic appearance of untreated contralateral (A) and implanted (B) mouse hindlimb 2 months after implantation. Arrow points at the proximal stitch used to anchor the scaffold to the native EDL muscle (blue). (**d**) Representative images showing macroscopic appearance of contralateral untreated EDL muscle (A), negative control muscle (B) and implanted scaffold (C) 2 months after implantation and after tibialis anterior dissection. (**e**) Representative images showing macroscopic appearance of contralateral untreated (UNTD, left) and LatB-, DET- and SDS-implanted EDL muscles (right) 2 months after implantation. (**f**) Mean weight of contralateral untreated EDL muscles (UNTD) and LatB-, DET- or SDS-implanted muscles two months after implantation. Data are shown as mean ± s.e.m.; n = 8-7, each group. No significant differences were observed among the groups. (**g**) *Ex vivo* force measurement of untreated EDL muscles, and LatB-, DET- and SDS-implants two months after implantation. Data are shown as mean ± s.e.m.; n = 6, each group. No significant differences were observed among the groups. (**h**) Ratio of the maximal force generated *ex-vivo* from regenerated implants (LatB, DET and SDS) and contralateral untreated EDL muscles two months after implantation. Data are shown as mean ± s.e.m.; n = 6, each group. No significant differences were observed among the groups. (**i**) Representative confocal images of cross-sections from contralateral untreated EDL muscles, and LatB-, DET- and SDS-implanted scaffolds stained with bungarotoxin (BTX - red) and synaptophysin (SYP - green) two months after implantation. Nuclei were stained with dapi (blue) and phase contrast (PhC) is shown to highlight staining localization (scale bar: 10 µm). (**j**) Percentage of neuromuscular junctions in untreated and implanted scaffolds calculated as percentage of SYP/BTX double-positive cells. **P < 0.01; ***P < 0.001 each decellularised sample compared to untreated muscles.

**Figure 5:**
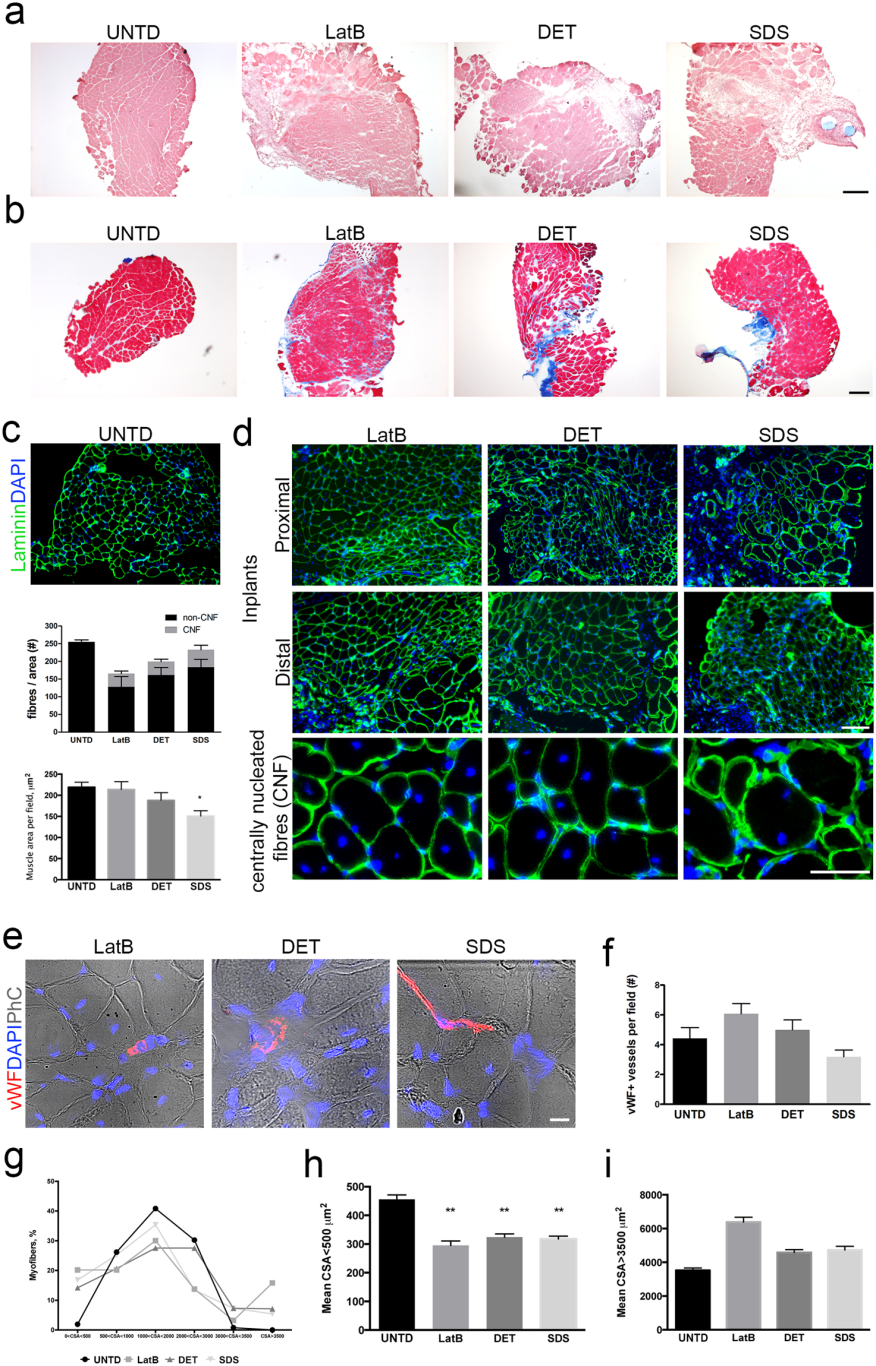
Decellularised skeletal muscles induce muscle regeneration in a mouse model of VML. (**a**) H&E staining of cross-sections from contralateral untreated EDL (UNTD) and the middle region of LatB-, DET- and SDS-implanted scaffolds two months after implantation. Scale bar: 250 µm. (**b**) Masson’s Trichrome staining of cross-sections from UNTD muscles and the middle region of LatB-, DET- and SDS-implanted scaffolds two months after implantation. Scale bar: 250 µm. H&E staining of cross-sections from UNTD muscles and LatB-, DET- and SDS-implanted scaffolds two months after implantation. Cross-sections showed centrally nucleated fibres, connective tissue and myofibres of variable size into the implanted scaffold. Scale bar, 50 µm. (**c**) Representative images of immunofluorescence analysis for laminin (green) in cross-sections from contralateral untreated EDL muscles, and (**d**) LatB-, DET- and SDS-implanted scaffolds, two months after implantation. Nuclei were stained with dapi (blue). Scale bar, 100 µm. Bottom line: higher magnifications to show centrally nucleated fibres (CNF). Scale bar, 50 µm. (**e**) Quantification of the number of fibres per field and respective contribution of CNF and non-CNF in UNTD muscles and implanted scaffolds, using images of laminin immunofluorescence staining. (**f**) Quantification of muscle area per field in UNTD muscles and LatB-, DET- and SDS-implanted scaffolds two months after implantation. *P < 0.05 compared to UNTD; one-way ANOVA and Tukey’s multiple comparison test; n = 4–6, each group. (**g**) Representative immunofluorescence images of cross-sections from LatB-, DET- and SDS-implanted scaffolds stained with for von Willebrand factor (vWF, red) two months after implantation. Nuclei were stained with dapi (blue) and phase contrast (PhC) is shown to highlight staining localization and tissue structure (scale bar: 10 µm). (**h**) Quantification of the number of vWF+ vessels in implanted scaffolds (n = 4–6). (**i**) Percentage of myofibres with cross-sectional area (CSA) less the 500 µm^2^, between 500 and 1000 µm^2^, between 1000 and 2000 µm^2^, between 2000 and 3000 µm^2^, between 3000 and 3500 µm^2^, or more than 3500 µm^2^ in UNTD muscles, LatB-, DET- and SDS-implanted scaffolds two months after implantation. n = 4–6, each group. (**j**) Mean cross-sectional area (CSA) of myofibres showing CSA less than 500 µm^2^ in UNTD muscles, LatB-, DET- and SDS-implanted scaffolds two months after implantation. Data are shown as mean ± s.e.m. **P < 0.01, compared to contralateral untreated muscles; data were analysed by using Kruskal-Wallis and Dunn’s multiple comparison test; n = 4–6, each group. (**k**) Mean cross-sectional area (CSA) of myofibres showing CSA more than 3500 µm^2^ in EDL contralateral untreated muscles (UNTD), LatB-, DET- and SDS-implanted scaffolds two months after implantation. Data are shown as mean ± s.e.m. Statistical analysis was performed by using Kruskal-Wallis and Dunn’s multiple comparison test and reported in Supplementary Table 1.

Support of *in vivo* muscle regeneration by the acellular muscles was tested using a mouse model of VML^37^, where the EDL muscles were resected and substituted with either LatB-, DET- or SDS-acellular scaffolds (Fig. 4a,b). All animals survived surgery without complication. Two months after implantation the macroscopic appearance of the site of implantation was free from scar tissue or inflammation (Fig. 4c). As expected^30^, no new tissue development or muscle regeneration were found between the two edges of the resected EDL in negative control animals (Fig. 4dB). In contrast, in all animals transplanted with decellularised scaffolds, the site of implantation showed tissue regeneration (Fig. 4dC,e). Artificial muscles were of similar size to contralateral untreated EDL muscles and had comparable weights (Fig. 4e,f). To investigate whether the regenerated tissues were functional, *ex-vivo* force generation analysis was performed. Interestingly, all the artificial muscles were able to contract and generate force with no significant differences observed between the three groups, despite showing partial restoration of muscle strength when compared to contralateral untreated EDL (Fig. 4g,h). Immunofluorescence analysis for the neural marker Tuj1 and bungarotoxin confirmed the presence of the nervous system in the implanted scaffolds two months after implantation (Supplementary Fig. 3), while bungarotoxin and synaptophysin co-staining demonstrated the presence of functional neuromuscular junctions in all implants (Fig. 4i). The percentage of synaptophysin/bungarotoxin double-positive NMJ were comparable in all implanted scaffolds, but significantly lower than untreated contralateral muscles (Fig. 4j). Moreover, histological examination revealed the presence of skeletal muscle tissue in the artificial muscle, and interestingly in the middle and distal regions of the implants (Fig. 5a–d, Supplementary Figs 4–5). Confirmation of muscle regeneration was given by the presence of centrally nucleated myofibres in the implants, which also showed correct deposition of laminin (Fig. 5c,d). The percentage of centrally nucleated fibres was comparable among the 3 implants, with no significant difference determined: LatB 28.76 ± 24.43, DET 19.71 ± 12.33 and SDS 19.38 ± 15.32 (n = 4–6) (Fig. 5e). Interestingly and in contrast to LatB- and DET-implants, mean muscular tissue area was reduced in SDS-implants, when compared to contralateral untreated muscles (Fig. 5d). Connective tissue deposition was evident mostly in the junction between scaffold and native muscle, as well as in the distal portion of the implanted scaffolds (Supplementary Fig. 5). In addition, all the artificial muscles showed clear evidence of vascularization throughout the length of the grafts, with a number of vWF+ vessels comparable to contralateral untreated muscles (Fig. 5g,h).

**Figure 6:**
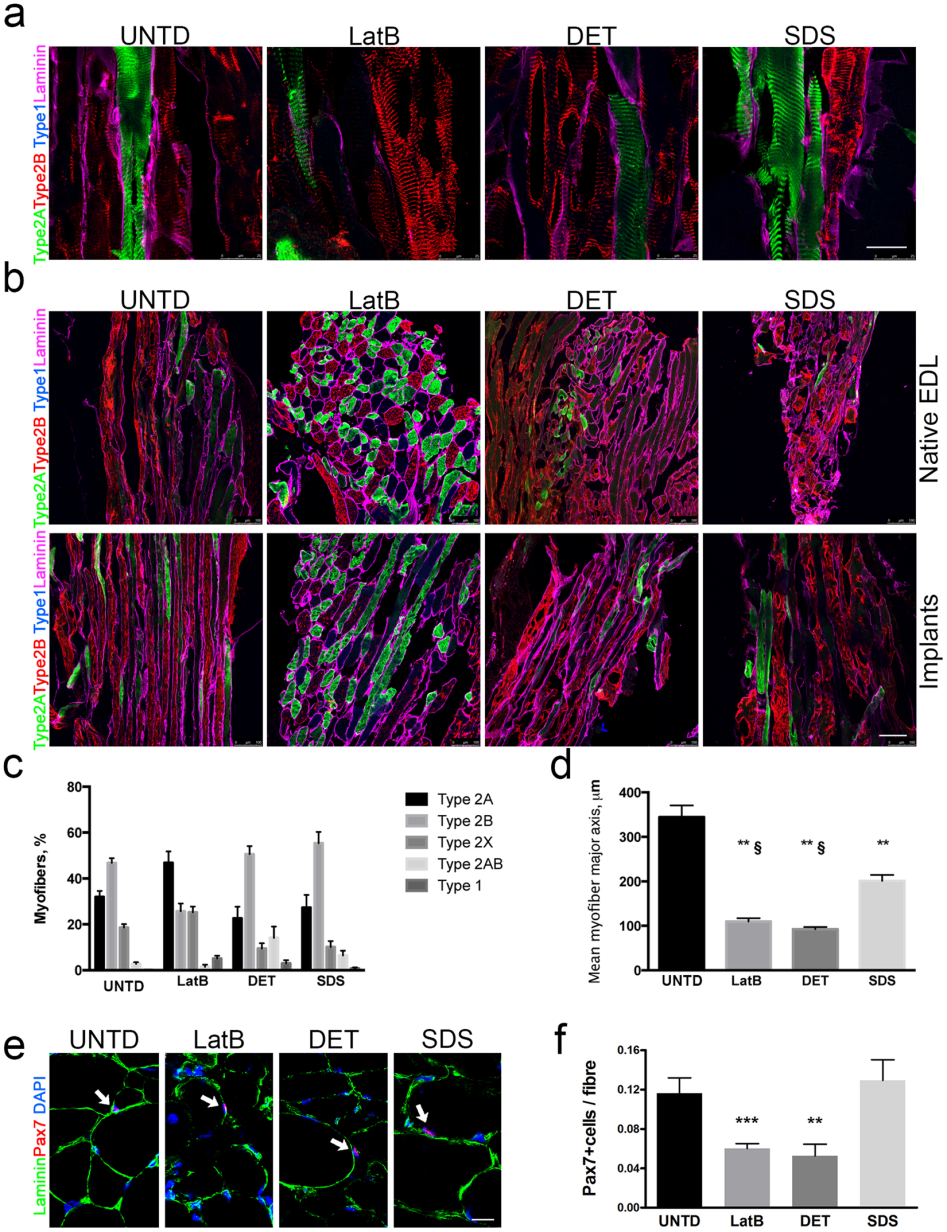
Characterization of myofibre type and length of LatB-, DET- and SDS-implanted scaffolds. (**a**) Representative z-stack confocal images of longitudinal sections from contralateral untreated EDL muscle (UNTD) and LatB-, DET- and SDS-implanted scaffolds two months after implantation. Laminin (magenta) and myosins recognising type 2 A (green), type 2B (red), type 2AB (green and red) and type 1 (blue) myofibres were identified by immunofluorescence. Scale bar: 25 µm. (**b**) Representative z-stack confocal images of longitudinal and cross-sectional sections from contralateral untreated EDL muscle (UNTD) and LatB-, DET- and SDS-implanted scaffolds two months after implantation. Type 2 A (green), type 2B (red), type 2AB (green and red), type 1 (blue), and type X (black) myofibres were identified together with laminin (magenta) staining. Scale bar, 100 µm. (**c**) Quantification of type 2 A, 2B, 2AB, type 1 and type X myofibres in contralateral untreated EDL muscle (UNTD) and LatB-, DET- and SDS-implanted scaffolds two months after implantation. Data are shown as mean ± s.e.m. Statistical analysis was performed by using two-way ANOVA and Tukey’s multiple comparison test and reported in Supplementary Table 2. (**d**) Quantification of the mean length of myofibre major axis in contralateral untreated EDL muscle (UNTD) and LatB, DET- and SDS-implanted scaffolds two months after implantation. **P < 0.01 compared to UNTD; ^**§**^P < 0.01 compared to SDS; one-way ANOVA and Tukey’s multiple comparison test; n = 4–6, each group. (**e**) Representative confocal images of sections from contralateral untreated EDL muscle (UNTD) and LatB-, DET- and SDS-implanted scaffolds two months after implantation; staining for Pax7 (red - arrows) and laminin (green) and dapi (blue). Scale bar: 10 µm. (**f**) Quantification of Pax7+ cells in sublaminar position per myofibre in contralateral untreated EDL muscle (UNTD) and LatB, DET- and SDS-implanted scaffolds two months after implantation. Data are shown as mean ± s.e.m. ***P < 0.001; **P < 0.01 compared to UNTD.

To investigate if regenerated skeletal muscles properly occurred into artificial muscles, morphometric analysis of fibre cross-sectional area (CSA) was performed. All decellularised scaffolds showed small or large fibres, compared to contralateral untreated muscles (Fig. 5i–k and Supplementary Fig. 6). No difference was observed among LatB-, DET- or SDS-implants regarding the mean CSA of small myofibres, however, LatB-muscles showed an higher mean CSA in bigger myofibres, when compared to DET and SDS-implants (Fig. 5j,k and Supplementary Table 2). Characterization of muscle fibres through myosin heavy chain profiling revealed that LatB-implants supported the development of fibre type 2A at the expenses of fibre type 2B, compared to DET and SDS scaffolds (Fig. 6). LatB-muscles also displayed a significant reduction in the percentage of fibre type 2B, when compared to contralateral untreated muscles (Fig. 6a–c). When myofibre dimension was quantified considering the major axis of the fibres, all the implanted scaffolds showed lower mean length of the major axis compared to contralateral untreated EDL muscles. Nevertheless, myofibres of SDS-implants were significantly “longer” when compared LatB- and DET-implants (Fig. 6d).

With the aim to understand if decellularised scaffold could also promote SCs homing, the number of sublaminal cells expressing the transcription factor paired box protein 7 (Pax7) in respect to the total myofiber number was quantified in LatB-, DET- and SDS-implants and compared to untreated contralateral EDL muscles. The presence of a pool of Pax7^+^ cells under the basal lamina of myofibres was evident in all implanted scaffolds. However, LatB- and DET- implants showed a lower number of Pax7+ cells compared to UNTD muscles and SDS-implants (Fig. 6e,f).

### Muscle stem cells can migrate along decellularised single myofibres

**Figure 7:**
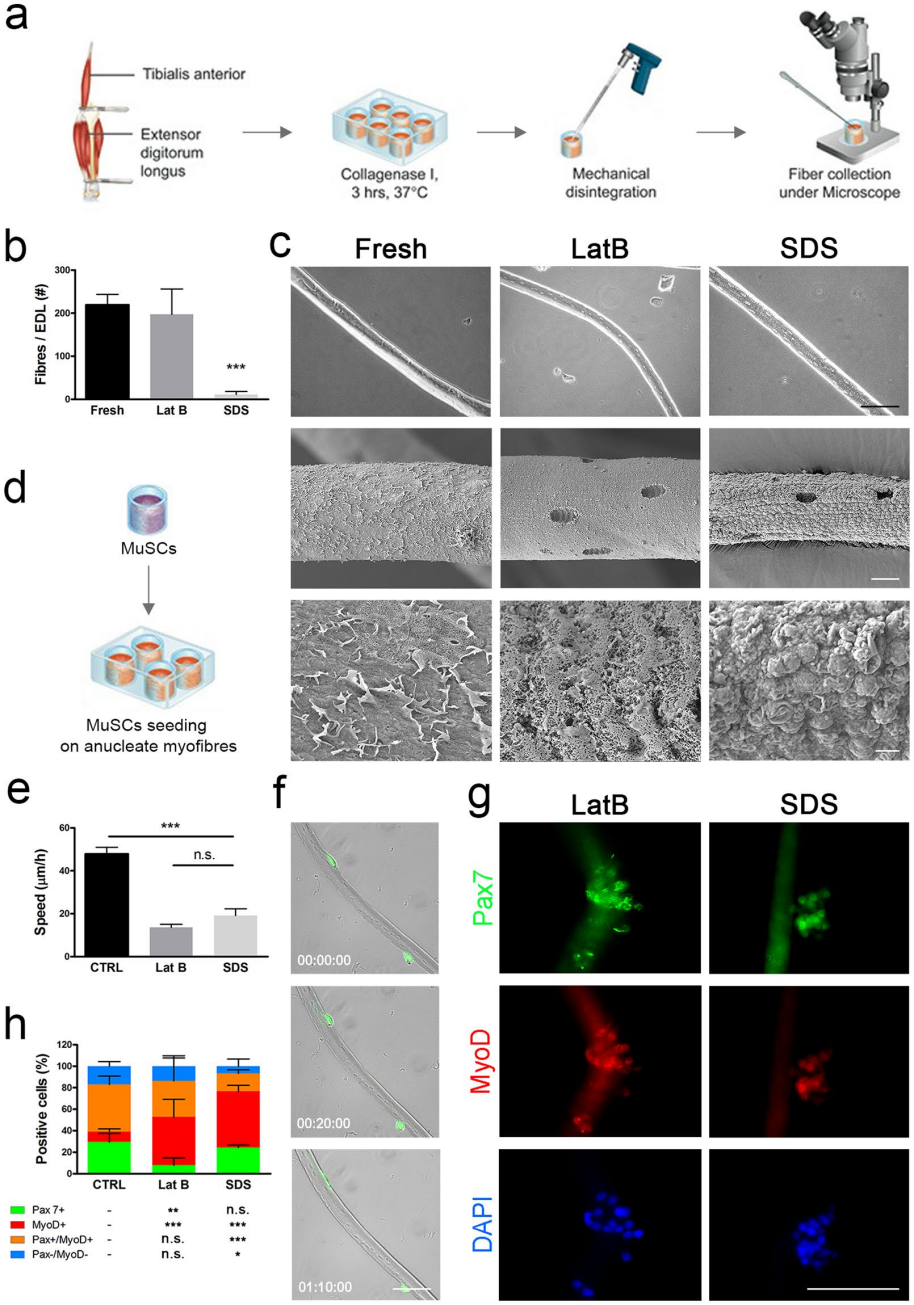
Isolation and seeding of anucleate myofibres. (**a**) Single myofibres were isolated from fresh or decellularised rat EDL digested in collagenase I and collected under the microscope. (**b**) Yield of fibres per EDL, ***p < 0.001 vs Fresh, t-test. (**c**) Scanning electron microscopy of single fresh and anucleate myofibres from LatB- and SDS-decellularised EDL. Scale bar: first row 100 µm, second row 10 µm, third row 1 µm. (**d**) Murine MuSCs were seeded on single myofibres and fresh EDL fibres were cultured to activate resident MuSCs. (**e**) Cellular migration speed along single fibres measured 24 hours post-seeding; ***p < 0.001, ANOVA. (**f**) Representative images of GFP+ MuSCs migrating on LatB-scaffold derived anucleate fibres, scale bar: 10 µm. (**g**) Immunofluorescence for MyoD and Pax7 of wild-type MuSCs seeded on LatB and SDS anucleate myofibres after 72 hours of culture, scale bar: 100 µm. (**h**) Subpopulations of cells according to positivity for Pax7 and MyoD after 72 hours of culture with statistical analysis showing significance in respect to control (CTRL). *p < 0.05; **p < 0.01; ***p < 0.001 vs CTRL, t-test.

Since muscle stem cells (MuSCs) are the major player of muscle regeneration, it is reasonable to speculate that MuSCs can migrate across the scaffolds and be involved in the artificial muscle regeneration. It is also have been showed that MuSCs present on single myofibres migrate and proliferate *in vitro*^35,38^. Therefore, we firstly evaluated the ability of MuSCs to recognise acellular myofibres, migrate and proliferate on their surface in a bi-dimensional *in vitro* cell culture system. To reach this aim, single fibres isolated from LatB and SDS acellular muscles were seeded with wild-type or GFP^+^ mouse MuSCs *in vitro* (Fig. 7a). DET was omitted as showing similar structure characteristics to LatB. Single fibres from LatB-acellular muscles were easier to isolated than from to SDS scaffolds, which produced very few intact anucleate fibres (Fig. 7b). Both LatB and SDS single fibres showed distinct appearance compared to those from normal freshly isolated ones, with notches evidencing the previous location of myonuclei (Fig. 7c). Interestingly, murine MuSCs were able to adhere to anucleate myofibres and migrate along their surface (Fig. 7d,f). Migration speed and mechanism (i.e. blebbing)^38^ were evaluated and compared to resident activated cells in mouse freshly isolated myofibres^38^. Cellular migration speed was reduced in both the anucleate myofibre preparations, when compared to activated MuSCs on freshly isolated myofibres (Fig. 7e). Nevertheless, analysis of movement of cells along the length of the fibres indicated comparable diffusive movement among the 3 conditions, showing similar cellular displacement along the fibre in the first 700 minutes of migration (Supplementary Fig. 7). This was associated to comparable blebbing dynamics (extension, stabilisation and retraction), lifespan, shape and size between fresh and anucleate seeded myofibres (data not shown). Next to their ability in activating cell migration with blebbing formation, LatB- and SDS-anucleate myofibres also supported MuSCs proliferation, reproducing what normally occurs in freshly isolated single fibres. Moreover, SDS-myofibres showed a higher ability to preserve cells expressing the key transcription factor Pax7, compared to LatB-anucleate myofibres (Fig. 7g,h).

### Migration of SCs in a decellularised skeletal muscle require fibroblasts

Despite MuSCs showed the ability to be cultured on single acellular fibres *in vitro*, we also evaluated their potential when cultured into 3D decellularised muscle. Indeed, the more complex and constrained culture environment that MuSCs found when cultivated into acellular matrix, in respect to the single fibre culture system, may influence MuSC behaviour. Moreover, this *in vitro* model better mimicked the hypothetical *in vivo* condition, in which myogenic cells had to migrate across the implanted scaffolds for its repopulation. We decided to perform the following studies only on SDS-scaffold based on the similar regenerative potential observed among of the three scaffolds *in vivo* and the results obtained in the single anucleated fibre experiments *in vitro*. Firstly, murine SCs were isolated and amplified as MuSCs only for 1 passage in proliferating medium, characterized before seeding into the scaffolds. As expected, stripped SCs grew in culture as colonies and, when dissociated as single cells, MuSCs were able to be expanded in proliferating supporting medium and to differentiate into myotubes in culture (Supplementary Fig. 8a). After two passages of expansion and just before seeding, MuSCs were characterized in terms of proliferation and expression of myogenic markers (Supplementary Fig. 8b,c). The majority of MuSCs was proliferating and showed high expression of Pax7 (71%) and the myogenic regulatory factor MyoD (75%). In contrast, only a small percentage of cells expressed the early differentiating transcription factor Myogenin (MyoG, 14%) and the late differentiating protein embryonic myosin heavy chain (eMyHC, 9%; Supplementary Fig. 8c). In line with published work^39^, the majority of Pax7^+^ cells co-expressed MyoD (89%; Supplementary Fig. 8c). In agreement with this fact and the low percentage of early differentiating cells (i.e. MyoG^+^), the majority of MyoD^+^ cells was still expressing Pax7 (80%, Supplementary Fig. 8c). Moreover, almost all the cells expressing Pax7 or MyoG were in a proliferating phase (93% and 85%, respectively; Supplementary Fig. 8c). Altogether, these results indicate that the MuSC cultures, that were used to repopulate the scaffolds, are constituted of proliferating myogenic cells that retained expression of the stem cell marker Pax7. Having confirmed their myogenic potential, we tested the ability of MuSCs to repopulate the decellularised SDS-scaffold.

**Figure 8:**
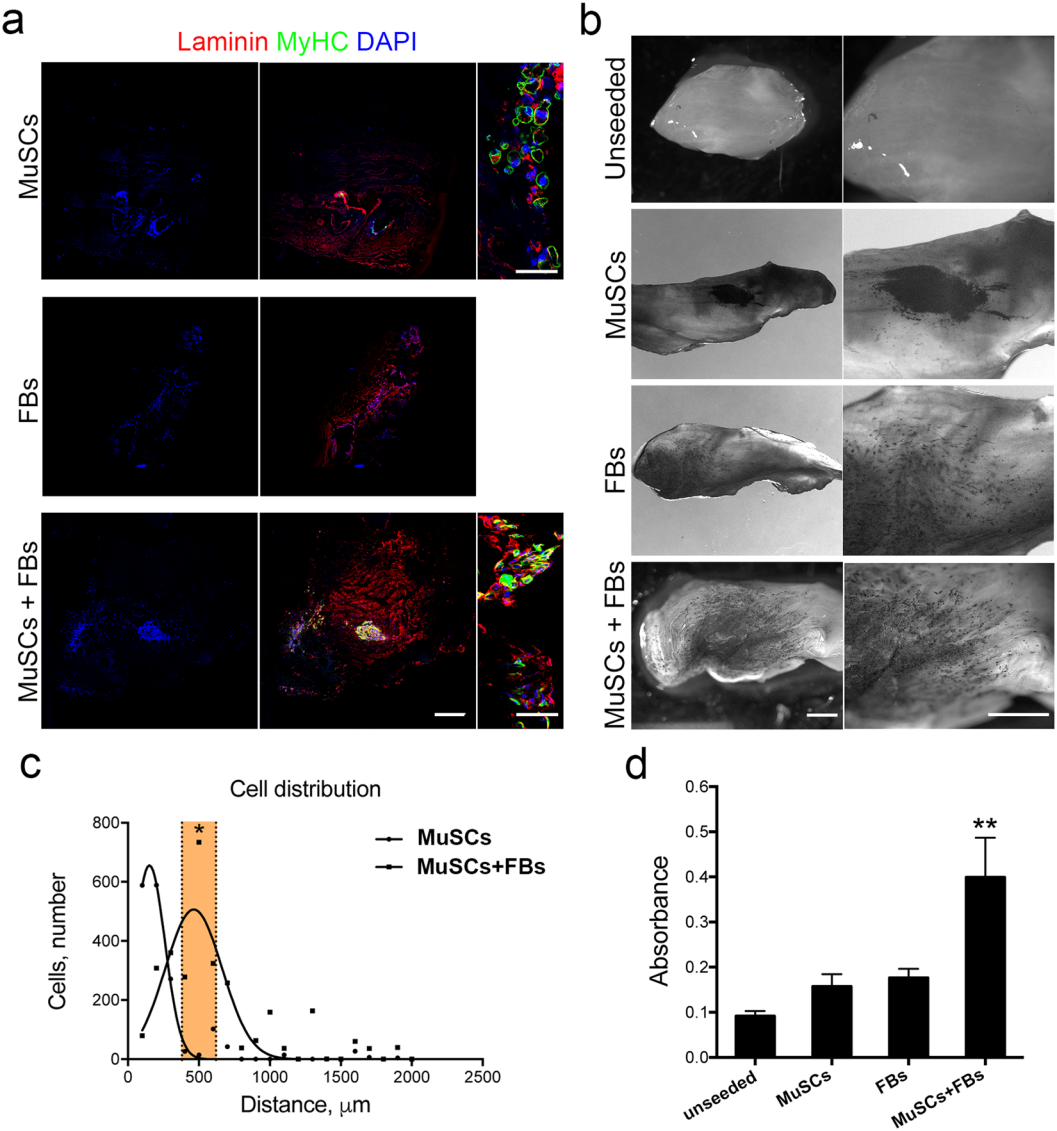
*In vitro* colonization of acellular scaffold using MuSCs and FBs. (**a**) Representative scan images of scaffold cross-sections seeded with MuSCs, FBs or MuSCs-FBs analysed by immunofluorescence analysis using anti-laminin (red) and anti-MyHC antibodies (green). Laminin was used to identify the entire cross section of the scaffolds. Nuclei were stained with Hoechst (blue). Scale bar: 500 μm. (**b**) Representative images acquired at stereomicroscope of MTT assay performed on unseeded or seeded scaffolds cultured with MuSCs, FBs or MuSCs-FBs. Scale bar: 1 mm. (**c**) Analysis of cell distribution in scaffold cross-sections seeded with MuSCs or MuSCs-FBs. *P < 0.05, one-way ANOVA and Tukey’s multiple comparison test; (**d**) Availability assay by MTT quantification. Data are shown as mean ± s.e.m. of six independent replicates. **P < 0.01; one-way ANOVA and Tukey’s multiple comparison test.

Interestingly, when MuSCs were injected into SDS- scaffolds and cultured *in vitro* for 7 days, a number of multinucleated myotubes expressing eMyHC were present at the injection site (Fig. 8a). These results indicated the ability of MuSCs to maintain the myogenic commitment and to undergo late muscle differentiation when cultivate into 3D acellular scaffolds (Fig. 8a). However, overall scaffold repopulation with MuSCs was unsuccessful, since cells were found only at the area of injection (Fig. 8). This was clearly different from the *in vivo* finding in which host cells repopulated the all implanted scaffolds and it was probably related to the fact that multiple cells orchestrate organization of scaffold colonization after transplantation. In particular, muscle fibroblasts (FBs) are known to play a key role in ECM remodelling and to regulate SC proliferation and differentiation during muscle regeneration^28,31,40,41^. Based on this established concept, we hypothesized that fibroblasts could be responsible of the efficient regeneration observed *in vivo* and they could therefore improve scaffold repopulation and myogenesis when seeded into the acellular muscles together with MuSCs *in vitro*. FBs were therefore isolated from mouse EDL and expanded in culture before being used for seeding experiments. Primary FBs displayed typical morphology and expressed vimentin and the nuclear factor Tcf4, but were negative for α-SMA expression (Supplementary Fig. 8d)^35,42^. Moreover, the majority of the cells were proliferating and did not expressed desmin, confirming the absence of contaminating myogenic cells into the FBs primary culture (Supplementary Fig. 8d).

To elucidate the influence of FBs on scaffold repopulation and myogenesis, a comparison of SDS scaffolds seeded with MuSCs or FBs or the combination of both MuSCs and FBs was performed after 7 days of culture (Fig. 8). Firstly, scaffold repopulation and cell migration from the site of injection were evaluated. FBs migrated readily within the scaffold and clearly affected the colonization of myogenic cells, which was strongly improved when MuSCs were seeded together with FBs. This was highlighted both by immunofluorescence (Fig. 8a,c) and identification of viable cells with MTT (Fig. 8d). Furthermore, differentiated myogenic cells (eMyHC-positive) were completely absent when FBs were seeded alone (Fig. 8a). Since FBs have been shown also to improve terminal differentiation of newly formed fibres *in vivo*^42–44^, SDS scaffolds seeded with MuSCs or MuSCs-FBs were analyzed in terms of cross-sectional area (CSA) of multinucleated myotubes. As observed *in vivo*^42–44^, myogenic cells infiltrating into acellular scaffolds generated large myotubes in presence of FBs (Supplementary Fig. 9). The maturation of myotubes after 7 days in 3D culture was further demonstrated by the spontaneous twitching of myotubes present in SDS scaffolds seeded with MuSCs and FBs (Supplementary Movie 1).

**Figure 9:**
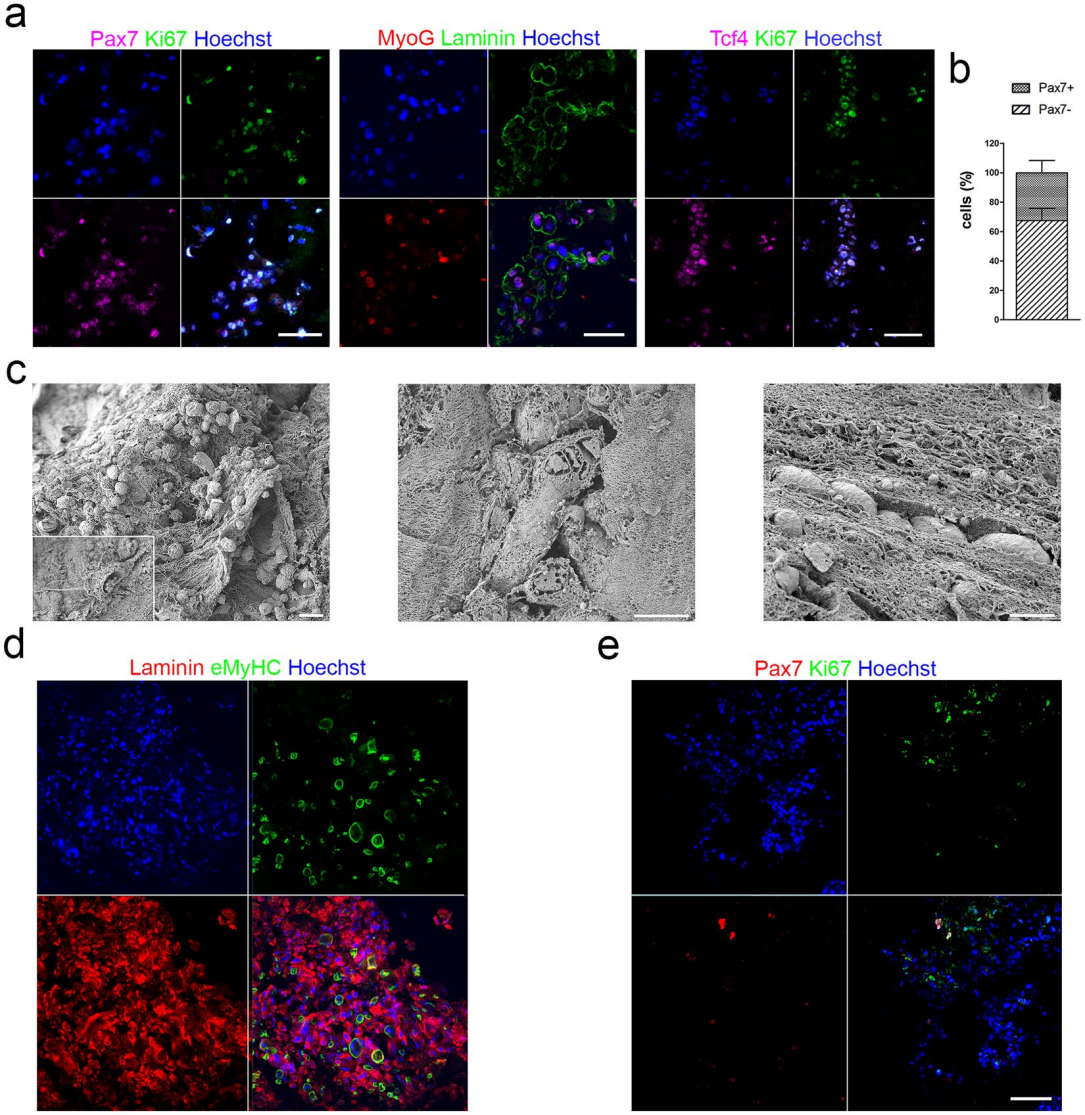
Characterization of SDS scaffolds repopulated with MuSCs and FBs. (**a**) Immunofluorescence analysis of scaffold cross-sections seeded with MuSCs-FBs and cultured for one week by using Pax7 (magenta) and ki67 (green), or MyoG (red) and laminin (green), or Tcf4 (magenta) and ki67 (green). Nuclei were stained with Hoechst (blue). Scale bar: 100 μm. (**b**) Percentage of Pax7-positive and negative cells in co-cultured scaffolds after one week. (**c**) SEM analysis showing cell-ECM interaction in SDS scaffolds seeded with MuSCs-FBs and cultured for one week. The insert show a single cell spreading its projections to the ECM. Scale bar: 20 μm left panel; 10 μm middle and right panel. (**d**) Immunofluorescence analysis of scaffold cross-sections seeded with MuSCs-FBs and cultured for two weeks by laminin (red) and eMyHC (green). Nuclei were stained with Hoechst (blue). Scale bar: 50 μm. (**e**) Immunofluorescence analysis of scaffold cross-sections seeded with MuSCs-FBs and cultured for two weeks by Pax7 (red) and ki67 (green). Nuclei were stained with Hoechst (blue). Scale bar: 50 μm.

Starting from these data, SDS scaffolds cultivated with MuSCs-FBs were further analysed for the presence of FBs, myogenesis and cell proliferation (Fig. 9). Seven days after culture, proliferating stem cells (ki67^+^Pax7^+^), committed myogenic cells, differentiated cells and myotubes (MyoG^+^) were found in SDS scaffolds, as showed by immunofluorescence analysis (Fig. 9a). Moreover, a population of Tcf4^+^ cells was also detected (21.8 ± 24% on total number of cells), some of which were proliferating (ki67^+^; Fig. 9a). The percentage of Pax7^+^ stem cells calculated on the total number of Tcf4^−^ cells was 67.5 ± 23.3% (Fig. 9b). Scanning electron microscopy (SEM) analysis revealed complex network of interaction created between seeded cells and ECM and confirmed the presence of multinucleated myotubes embedded into the ECM of the scaffolds. Furthermore, cells appeared to align following the 3D organization of the ECM that formed a track generated with the decellularisation process that removed the majority of the pre-existing myofibres (Fig. 9c). To understand if decellularised scaffolds could also allow cell maintenance and support myogenesis for a longer period of culture, MuSCs co-seeded with FBs were grown in culture for 14 days (i.e. 2 days in proliferating media and 12 days in differentiating media). Interestingly, prolonged cell culture increased scaffold repopulation, permitted the maintenance of myotube and the Pax7^+^ cell population and preserved cell proliferation (Fig. 9d,e).

## Discussion

VML represents still a clinical challenge and a definitive therapeutic solution has not yet been described. ECM components have been shown to play important role for promoting proper muscle regeneration and maintenance of SC stemness^35,39,45,46^. Decellularised matrices have the advantage of mimic architecture and organization of the native tissue and ideally presents the same biochemical composition, cell homing activation, biomechanical and angiogenic properties of the tissue of interest^31,40,41^. Since the decellularisation procedure can alter the ECM, compromising its biochemical, biomechanical and structural properties, we optimized and compared three different protocols previously published on non-perfusion decellularisation for skeletal muscles^29–33^. We obtained three different homogenous decellularised skeletal muscles that showed comparable ECM components, but with different preservation of myofibre structure. LatB and DET-scaffolds maintained fine myofibre structure and could therefore described as enucleated instead of decellularised. Myofibres were largely absent following SDS treatment, which was mainly constituted by ECM. For reasons of clarity and direct comparison to other published studies, scaffolds were still referred to as decellularised. Although the importance of ECM components has been demonstrated for *in vivo* scaffold repopulation by host cells^5^, the possible role of sarcolemmal proteins and other components remains under debate. Despite the similarity with LatB-acellular muscles discussed above, DET-scaffolds showed almost complete absence of all the proteins located at the sarcolemma analysed. Interestingly, SDS-scaffolds partially preserved α- and β-dystroglycans, suggesting that sarcolemmal proteins could still be retained, probably through the connection to the ECM components.

Besides preservation of proteins and tissue architecture, efficient regeneration depends also on the ability of the graft to maintain specific chemokines known to be important in promoting angiogenesis and cellular migration while reducing inflammation to facilitate repopulation and local regeneration^5,27^. Among those, we focused on VEGF and IGF, which are respectively involved in promoting vessel formation and in supporting muscle growth, regeneration and maintenance^47^. LatB-, DET- and SDS-acellular muscles all showed maintenance of IGF1. Differently, only DET- and SDS-acellular muscles showed the maintenance of VEGF, while LatB scaffolds did not preserve a detectable amount of VEGF, showing reduce ability to attract vessels in CAM assay. This suggest that other mechanisms rather than VEGF could be involved in the promotion of vessel formation by LatB-scaffolds upon implantation.

Despite the varying degrees of muscle micro-architecture preservation in our scaffolds, we found extensive muscle regeneration two months after implantation in all implanted scaffolds. We observed correct deposition of matrix around newly formed myofibres, formation of functional myofibres, together with blood vessels, invasion of nerves and functional NMJ, with no differences among the three different scaffolds. Importantly, all the implanted scaffolds allowed the homing of SCs –i.e Pax7^+^ cells in sublaminal position. Functional recovery of the treated muscles was confirmed by the ability of artificial muscles to contract and generate force in *ex vivo* physiological activity tests. Despite SDS-regenerated muscles showed reduced muscle area when compared to the other implants, we did not observe functional differences in the recovery of the transplanted muscle. All artificial muscles displayed altered myofibre CSA and myofibre major axis length together with increased presence of connective tissue, when compared to untreated contralateral EDL muscles. These features, together with the reduced number of functional NMJ observed in the artificial muscles, could explain the reduction in force production observed in the implants, when compared to untreated contralateral muscles. At deeper investigation, *in vivo* muscle remodelling activated by LatB-, DET- and SDS-implants gave rise to muscles with different characteristics, which could have implications on large VML repair. SDS-muscles displayed a myofibre type ratio similar to untreated EDL muscles together with bigger myofibre major axis length, when compared to LatB- and DET-implants. In addition, the SDS-regenerated muscles were the only implants in which the number of SCs was found to be comparable to untreated contralateral EDL muscles. Different hypotheses could explain the contrasting results obtained with LatB-, SDS- and DET-scaffolds. For example, it is possible that the presence of the cytoplasm of myofibres in LatB- and DET-scaffolds caused a mechanical obstacle during scaffold repopulation, which did not allow the right orientation of the newly formed myofibres and/or myofibre type profile, as well as correct restoration of SC pool. Based on these findings, we can conclude that myofibre preservation in decellularized skeletal muscles do not improve host cell response. Instead, the use of a harsher decellularization that removes myofibre content but maintaining the ECM 3D organization and composition –i.e. SDS-scaffolds, was found to be more efficient in promoting muscle regeneration and cell homing *in vivo*. In SDS-implants, the presence of the remodelling ECM can allow the formation of better aligned myofibres with right specification, together with proper SC pool preservation, which can potentially permit further cycles of muscle regeneration.

Different studies demonstrate that upon implantation, acellular tissues can be invaded by host cells and be remodelled, allowing host progenitor and stem cells to populate the new matrix^5,25,27,31,40,48^. Muscle-derived scaffolds obtained with protocols similar to those described in this study have shown to activate an immune modulatory host response that leads to scaffold degradation and new tissue formation. According with this and based on the histology observed in artificial muscles, we can hypothesize that this is also happening in our implanted scaffolds which have been remodeled and replaced by new tissue. How the enucleated myofibers are degraded –i.e. in LatB- and DET-implants, and the possible preservation of donor extracellular matrix components remain matters of further investigation. The degradation of the ECM-derived scaffolds has shown to have a positive influence on tissue regeneration since it induces the release of bioactive molecules that modulate macrophage polarization phenotype and activate local stem cells^49–51^. Despite further analysis need to be done to clarify the role of different cell types in the development of artificial muscles *in vivo*, the presence of SCs in implanted muscles and the *in vitro* results demonstrated the involvement of MuSCs in the promotion of muscle regeneration observed in LatB-, DET- and SDS-artificial muscles. The importance of MuSCs in promoting tissue regeneration, vascularization and innervation of decellularised matrix implants revealed in our study have been suggested also from others^5,25,27,31,33,40,48,52,53^. According to *in vivo* results, our *in vitro* studies demonstrated the ability of acellular muscles to support MuSCs adhesion, proliferation, maintenance of Pax7 expression and differentiation. Despite MuSCs were able to migrate along decellularised isolated fibers, the more complex 3D environment required the presence of fibroblasts to achieve MuSC colonization and myogenesis into acellular muscles. Fibroblasts have a crucial role on secreting key components of the extracellular matrix, such as collagen VI and fibronectin, which are essential for SC maintenance^35,46^. Moreover, fibroblasts also directly influence the dynamics of MuSCs during regeneration and their absence lead to the inability of MuSCs to proliferate and efficiently regenerate the site of injury following cardiotoxin injection^43^. Such cross-talk between MuSCs and FBs was also evident in our analysis, since the two cell population do not undergo to a selection process under the specific culture conditions used, respecting approximatively the ratio used at the seeding. We can therefore speculate that the process by which our scaffolds promote *in vivo* muscle progenitor migration involved various pathways, but it maybe ultimately guided by fibroblasts.

Xenogeneic scaffolds derived from other tissues rather than skeletal muscle coupled with physical therapy have been shown to partially ameliorate force outcome of a cohort of patients affected by VML^24^. A recent study demonstrate that acellular matrix derived from small-intestine submucosa and urinary bladder matrix failed to recover in a porcine model of VML^54^. Therefore, it still remains a matter of discussion whether the use of tissue specific acellular muscles could improve the overall clinical outcome of treated patients. Among limitations in applying acellular skeletal muscles scaffolds in clinic, decellularisation procedure represents *per se* a challenging aspect for this tissue, since its great thickness represent a limiting step for obtaining good quality and reproducible scaffolds. Indeed, studies involving the use of acellular skeletal muscle only refer to their application in animal models of VML. The majority of these studies used acellular muscles obtained by immersion protocols of decellularisation and often in combination with cell therapy^25,40,41,48,52,55–57^. In particular, a recent study reported the ability of MuSCs in combination with muscle resident cells (MRCs) pre-seeded in acellular skeletal muscles to restore structure and function of muscles with acute and chronic VML injuries, which can be further improved by a regimen of exercise^25^. Despite the VML model used in our study differed from what reported above^25^, the added value of our work resides in the demonstration that acellular skeletal muscles obtain with our perfusion protocols allow the development of functional artificial muscles without the implementation of donor cells in severe VML model and also in a xenogeneic model. Considering the well-known limitations of cell therapy approaches, the consequence that this aspect can have for future clinical application of such scaffolds as device to treat VML is extremely important. Devices based on decellularised muscles do not require expensive and potentially dangerous autologous cell harvesting and expansion and therefore facilitate clinical translation of VML treatment. Moreover, the ability of acellular muscles not coupled with cell therapy to promote functional muscle regeneration has been poorly investigated. Zhang *et al*. reported absence of innervation and scattered muscle formation upon implantation of acellular muscles derived by perfusion methods of decellularisation^26^. Differently, we demonstrated with three different perfusion methods of decellularisation that acellular muscles can promote extensive myogenesis, as well as functional innervation. Moreover, we show here for the first time, by comparing different method of decellularization, that myofibre preservation in decellularized skeletal muscles may be irrelevant for regeneration, which appears to be mainly driven by ECM 3D organization and composition. In conclusion, we strongly believe that these findings are of great importance in the design of new therapies based on tissue-engineering technology and may influence the design of scaffolds for engineering skeletal muscle tissue, as well as in the future application of decellularised muscles as *in vitro* models to investigate cell interplay during myogenesis.

## Electronic supplementary material

Supplementary Material

Supplementary Movie 1
