# Supplementary material for: Decellularised skeletal muscles allow functional muscle regeneration by promoting host cell migration
Source: Sci Rep. 2018 May 30;8:8398. doi: 10.1038/s41598-018-26371-y (PMC5976677; doi:10.1038/s41598-018-26371-y)
Supplement: Supplementary file 1 — Supplementary Material [file 41598_2018_26371_MOESM1_ESM.docx]

**Decellularised skeletal muscles allow functional muscle regeneration by promoting host cell migration.**

Anna Urciuolo^1,2,#^, Luca Urbani^1,3,4,#^, Silvia Perin^1^, Panagiotis Maghsoudlou^1^, Federico Scottoni^1^, Asllan Gjinovci^1^, Henry Collins-Hooper^5^, Stavros Loukogeorgakis^1^, Athanasios Tyraskis^1^, Silvia Torelli^1^, Elena Germinario^6^, Mario Enrique Alvarez Fallas^1^, Carla Julia-Vilella^1^, Simon Eaton^1^, Bert Blaauw^2,6^, Ketan Patel^5^, Paolo De Coppi^1*^.

^1^ Great Ormond Street Institute of Child Health, University College of London, London, WC1N 1EH, United Kingdom

^2^ Venetian Institute of Molecular Medicine, Via Orus 2, 35128, Padua, Italy

^3^ Institute of Hepatology London, Foundation for Liver Research, 111 Coldharbour Lane, London, SE5 9NT

^4^ Faculty of Life Sciences & Medicine, King’s College London

^5^ School of Biological Sciences, University of Reading, RG6 6UB, UK

^6^ Department of Biomedical Sciences, University of Padova, Italy

^#^ These authors contributed equally to this work

^*^ Corresponding author:

Paolo De Coppi, MD, PhD

NIHR Professor of Paediatric Surgery

Nuffield Professor of Paediatric Surgery

Head of Stem Cells & Regenerative Medicine Section, DBC, UCL

Surgery Offices

UCL Institute of Child Health

[30 Guilford Street](https://webvpn.gosh.nhs.uk/+CSCO+0h75676763663A2F2F636265676E79312E746266752E6175662E6878++/owa/UrlBlockedError.aspx)

[London WC1N 1EH](https://webvpn.gosh.nhs.uk/+CSCO+0h75676763663A2F2F636265676E79312E746266752E6175662E6878++/owa/UrlBlockedError.aspx)

Tel: [020 7905 2641](tel:020%207905%202641)(Administrator)

Fax: [020 7404 6181](tel:020%207404%206181)

email: [p.decoppi@ucl.ac.uk](mailto:p.decoppi@ucl.ac.uk)

**Supplementary Figure 1. Definition of the cycles necessary to allow good rate of decellularisation.** Quantification of DNA content and H&E analysis of freshly isolated skeletal muscle and LatB-, DET- and SDS-decellularised muscles after one, two or three cycles of treatment (cycle 1, cycle2 and cycle3, respectively). Arrows point at residual nuclei in treated tissues. Scale bar: 100 µm. Data are shown as mean ± s.d. of three independent replicates; * *P* < 0.05, † *P* < 0.01, ‡P < 0.001 test T of each decellularised sample compared to fresh muscles; n = 6-10, each group.

**Supplementary Figure 2. Immunofluorescence analysis of decellularised skeletal muscles.** Representative images of Immunofluorescence analysis for collagen I, dystrophin, α-dystroglycan and β-dystroglycan (all red) in cross-sections from freshly isolated rat skeletal muscle (fresh), LatB-, DET- and SDS-acellular muscles. Nuclei were stained with dapi (blue). Scale bar: 50 µm.

**Supplementary Figure 3. Innervation of implanted scaffolds.** Representative images of cross-sections from LatB-, DET- and SDS-implanted scaffolds stained with bungarotoxin (BTX - red) and Tuj1 (green) two months after implantation. Relative phase contrast images are also shown. Nuclei were stained with dapi (blue). Scale bar: 100 µm.

**Supplementary Figure 4. Histological characterization of transplanted decellularised muscles. a.** H&E staining of cross-sections from contralateral untreated EDL muscle (UNTD) and LatB-, DET- and SDS-implanted scaffolds two months after implantation. Cross-sections from the proximal edge of the implanted EDL, toward the proximal to the distal part of implanted scaffolds are shown. Scale bar: 250 µm. **b.** Trichrome staining of cross-sections from contralateral untreated EDL muscle (UNTD) and LatB-, DET- and SDS-implanted scaffolds two months after implantation. Cross-sections from the proximal edge of the implanted EDL, toward the proximal to the distal part of implanted scaffolds are shown. Scale bar: 250 µm.

**Supplementary Figure 5. Decellularised skeletal muscles induce muscle regeneration in a mouse model of VML. a.** H&E staining of cross-sections from contralateral untreated EDL muscle (UNTD) and LatB-, DET- and SDS-implanted scaffolds two months after implantation. Cross-sections showed centrally nucleated fibres, connective tissue and myofibres of variable size into the implanted scaffold. Scale bar: 50 µm. **b.** Trichrome staining of cross-sections from contralateral untreated EDL muscle (UNTD) and LatB-, DET- and SDS-implanted scaffolds two months after implantation. Myofibres were find also in the area with significant presence of connective tissue (upper panels). Myofibres of increased size were present into the implanted scaffold when compared to contralateral untreated EDL muscles (bottom panels). Scale bar: 50 µm. **c.** Masson’s Trichrome staining revealing connective tissue in the distal area of LatB-, DET- and SDS-implanted scaffolds two months after implantation. Contralateral untreated muscle (UNTD) showed normal collagen deposition. Scale bar: 50 µm.

**Supplementary Figure 6. Cross-sectional area analysis of LatB-, DET- and SDS-implants two months after surgery. a.** Myofibre cross-sectional area (CSA) in contralateral untreated EDL muscle (UNTD), LatB-, DET- and SDS-implanted scaffolds two months after implantation. Data are shown as mean ± s.e.m. ** P < 0.01 compared to UNTD; **§** P < 0.01 compared to LatB; **†** P < 0.01 compared to DET; one-way ANOVA and Tukey’s multiple comparison test; n =4-6, each group. **b.** Mean cross-sectional area (CSA) of myofibres showing CSA between 500 and 1000 µm^2^ in contralateral untreated EDL muscle (UNTD), LatB-, DET- and SDS-implanted scaffolds two months after implantation. No significant differences were observed among the different groups. n =4-6, each group. **c.** Mean cross-sectional area (CSA) of myofibres showing CSA between 1000 and 2000 µm^2^ in contralateral untreated muscles (UNTD), LatB-, DET- and SDS-implanted scaffolds two months after implantation. No significant differences were observed among the different groups. n =4-6, each group. **d.** Mean cross-sectional area (CSA) of myofibres showing CSA between 2000 and 3000 µm^2^ in UNTD, LatB-, DET- and SDS-implanted scaffolds two months after implantation. ** P < 0.01 compared to untreated muscles; Kruskal-Wallis and Dunn’s multiple comparison test; No significant differences were observed among the treated groups. n =4-6, each group.

**Supplementary Figure 7. Characteristics of MuSC motion.** The dots denote the data points and the blue curve is the curve of best fit, which is linear in all images. Mean squared displacement of mouse MuSCs along the x-axis of freshly isolated myofibres (CTRL) or MuSCs seeded on anucleate myofibres isolated from LatB- and SDS-decellularised EDL muscles.

**Supplementary Figure 8. Characterization of MuSCs and FBs. a.** Colonies of rounded MuSCs can be observed on gelatin coated dishes (left panel). MuSCs are able to differentiate in myotube when cultivated in differentiating media (right panel). Scale bar, 50 μm. **b.** Representative images of amplified MuSCs and labeled with antibody for Pax7 (green), Ki67 (grey), MyoD (grey), MyoG (green) and eMyHC (green). Nuclei were stained with Hoechst (blue). Scale bar: 100 μm. **c.** Quantification of ki67-, Pax7-, MyoD-, MyoG- and eMyHC-expressing cells in the MuSC culture, calculated on the total amount of cells and expressed as percentage (left upper panel). Quantification of MyoD expression in Pax7^+^ cell population (right upper panel). Quantification of Pax7 expression in MyoD^+^ cell population (left bottom panel). Quantification of proliferating (Ki67^+^) cells into the Pax7^+^ or MyoG^+^ cell populations (right bottom panel). Data are shown as mean ± s.e.m. of three independent replicates. ** P < 0.01; unequal variance Student’s test. **d.** Representative bright field image of cultured FBs. Scale bar: 50 μm. Immunofluorescence image showing expression of vimentin (green) and absence of desmin (red). Representative confocal image of immunofluorescence analysis for α-SMA (red), Ki67 (grey), Tcf4 (green) of FBs primary culture. Nuclei were stained with Hoechst (blue). Scale bar: 100 μm.

**Supplementary Figure 9. Myotube cross sectional area. a.** Representative H&E staining of multinucleated myotubes present into SDS scaffolds cultured with MuSCs or MuSCs-FBs. Scale bar: 50 μm. **b.** CSA quantification of multinucleated myotube present into SDS scaffolds cultured with MuSCs or MuSCs-FBs. Data are shown as mean ± s.e.m. of three independent replicates, ** *P* < 0.01; unequal variance Student’s test.

| Dunn’s multiple comparison test | Significant? | Summary (P) |
| --- | --- | --- |
|  |  |  |
| UNTD vs LatB | Yes | ** |
| UNTD vs DET | Yes | ** |
| UNTD vs SDS | Yes | * |
| LatB vs DET | Yes | * |
| LatB vs SDS | Yes | ** |
| DET vs SDS | No | ns |

**Supplementary Table 1. Statistical analysis of mean CSA analysis reported in Figure 5g.** Data were analysed by using Kruskal-Wallis and Dunn’s multiple comparison test; * P < 0.05; ** P < 0.01; ns: not significant. n = 4-6, each group.

| Tukey’s multiple comparison test | Significant? | Summary (P) |
| --- | --- | --- |
|  |  |  |
| Type 2A |  |  |
| UNTD vs LatB | Yes | ** |
| UNTD vs DET | No | ns |
| UNTD vs SDS | No | ns |
| LatB vs DET | Yes | ** |
| LatB vs SDS | Yes | ** |
| DET vs SDS | No | ns |
|  |  |  |
| Type 2B |  |  |
| UNTD vs LatB | Yes | ** |
| UNTD vs DET | No | ns |
| UNTD vs SDS | No | ns |
| LatB vs DET | Yes | ** |
| LatB vs SDS | Yes | ** |
| DET vs SDS | No | ns |
|  |  |  |
| Type 2X |  |  |
| UNTD vs LatB | No | ns |
| UNTD vs DET | No | ns |
| UNTD vs SDS | No | ns |
| LatB vs DET | Yes | ** |
| LatB vs SDS | Yes | ** |
| DET vs SDS | No | ns |
|  |  |  |
| Type 2AB |  |  |
| UNTD vs LatB | No | ns |
| UNTD vs DET | No | ns |
| UNTD vs SDS | No | ns |
| LatB vs DET | Yes | * |
| LatB vs SDS | No | ns |
| DET vs SDS | No | ns |
|  |  |  |
| Type 1 |  |  |
| UNTD vs LatB | No | ns |
| UNTD vs DET | No | ns |
| UNTD vs SDS | No | ns |
| LatB vs DET | No | ns |
| LatB vs SDS | No | ns |
| DET vs SDS | No | ns |

**Supplementary Table 2. Statistical analysis of myofibre type percentages reported in Figure 6c.** Data were analysed by using two-way ANOVA and Tukey’s multiple comparison test; * P < 0.05; ** P < 0.01; ns: not significant. n = 4, each group.

**Supplementary Movie 1. Spontaneous twitching of myotubes present in SDS scaffolds seeded with MuSCs and FBs.**
